# DNA methylome and transcriptome landscapes revealed differential characteristics of dioecious flowers in papaya

**DOI:** 10.1038/s41438-020-0298-0

**Published:** 2020-06-01

**Authors:** Ping Zhou, Xiaodan Zhang, Mahpara Fatima, Xinyi Ma, Hongkun Fang, Hansong Yan, Ray Ming

**Affiliations:** 1 College of Life Sciences, FAFU and UIUC Joint Center for Genomics and Biotechnology, Fujian Provincial Key Laboratory of Haixia Applied Plant Systems Biology, Fujian Agriculture and Forestry University, 350002 Fuzhou, Fujian China; 2 Fruit Research Institute, Fujian Academy of Agricultural Sciences, 350013 Fuzhou, Fujian China; 3 Department of Plant Biology, University of Illinois at Urbana-Champaign, Urbana, IL 61801 USA; 4 College of Agriculture, Fujian Agriculture and Forestry University, 350002 Fuzhou, Fujian China

**Keywords:** Epigenomics, Plant evolution

## Abstract

Separate sexes in dioecious plants display different morphology and physiological characteristics. The differences between the two sexes lie in their highly differentiated floral characteristics and in sex-related phenotype, which is genetically determined and epigenetically modified. In dioecious papaya (*Carica papaya* L.), global comparisons of epigenetic DNA methylation and gene expressions were still limited. We conducted bisulfite sequencing of early-stage flowers grown in three seasons (spring, summer and winter) and compared their methylome and transcriptome profiles to investigate the differential characteristics of male and female in papaya. Methylation variances between female and male papaya were conserved among three different seasons. However, combined genome-scale transcriptomic evidence revealed that most methylation variances did not have influence on the expression profiles of neighboring genes, and the differentially expressed genes were most overrepresented in phytohormone signal transduction pathways. Further analyses showed diverse stress-responsive methylation alteration in male and female flowers. Male flower methylation was more responsive to stress whereas female flower methylation varied less under stress. Early flowering of male papaya in spring might be associated with the variation in the transcription of *CpSVP* and *CpAP1* coinciding with their gene-specific hypomethylation. These findings provide insights into the sex-specific DNA methylation and gene expression landscapes of dioecious papaya and a foundation to investigate the correlation between differentiated floral characteristics and their candidate genes.

## Introduction

Dioecious species represent 6% of angiosperm species and 10% of land plant species^1^. Dioecy evolves from its hermaphroditic ancestor through gynodioecy or monoecy^1–3^. Sex determination genes regulate developmental process of floral organs and sex chromosomes ensure the stability of sex differences in dioecious species. Epigenetic reprogramming, in addition to genetic regulation, could also play important roles in sex determination of some flowering plants due to the phenomena that some flowering plants often have sex reversal under stress conditions.

Plants can perceive developmental or environmental stimuli and then carry out a variety of epigenetic alterations, including DNA methylation, histone modification and microRNA-dependent gene silencing, to reprogram the expression of floral genes. Epigenetic reprogramming might have a negative impact on the differentiation or development of stamen and carpel primordia, causing the abortion of reproductive organs and thereby the sex separation in flowering plants. Several epigenetic mechanisms of sex determination in some species were reported recently. In *Diospyros*, a small RNA targeting or methylated suppression of sex determinant was implicated^4,5^. Chemical hypomethylation in *Silene latifolia* and *Diospyros kaki* with the application of 5-azacytide or zebularine induced a striking sex reversion^4,6^. In melon, methylation alteration of sex-determinant genes resulted in sex differentiation^7^. These findings suggest that epigenetic regulation could also control the sex determination of some flowering plants.

Wild papaya is dioecy with male and female flowers in separate individuals. The sex of dioecious papaya is genetically determined by a pair of nascent XY sex chromosomes^8–10^. Sometimes low temperature induced male-to-hermaphrodite sex reversal in dioecious papaya, which was thought to be epigenetically modified results^11,12^. The genotype of sex chromosomes in female is XX, while that in male is XY, and the sex determination genes were located in the nonrecombining sex-determining region (SDR) of the Y chromosomes. For dioecious papaya, SDR refers to male-specific region of Y chromosome (MSY). High abundance of transposons and heterochromatin with intensive methylation confer sex-specific methylation pattern of SDR^13^. However, whether genome-wide sex-specific methylomes exist in the flowers of different sex forms remains unknown. If methylation variations do exist, could epigenetic modification of the genome reset the transcriptional expression of key genes to alter sex differentiation? It is also unclear whether the environmental factors could have an impact on shaping methylome landscapes.

In this study, based also on the whole-genome methylation sequencing, methylation profilings in both papaya male and female flowers were conducted to test whether the methylome variances exist in papaya flowers of different sexes and the correlation between them. From comparative methylome analyses, we found conserved methylation variances in both sexes and their diverse methylation alteration in response to stress. Combined transcriptomic evidences and phenotypic variances expand our understanding on papaya epigenome and transcriptome in relation to different sex types.

## Materials and methods

### Plant materials

Papaya variety “Zhonghuang” (dioecious, full-sib mating for several generations) was planted in the greenhouse of Fujian Agriculture and Forestry University. All papaya trees used in this study derived from the seeds of a single fruit harvested in 2015, which could minimize naturally existing genetic and epigenetic variances. We collected small inflorescences near the shoot apical meristem at noon and immediately preserved them on dry ice. The collection of spring flowers was conducted in April 2017 at noon with the average ambient temperature at 24 °C; summer flowers were collected in August when the average temperature is at 39 °C; winter flowers were collected in February 2018 with the average temperature at 10 °C. Average temperatures at 39 or 10 °C are considered unsuitable for the growth of papaya and the samples collected under these conditions were regarded as being grown under stress.

The early-stage flowers whose length was shorter than 1.5 mm were collected for DNA and RNA extraction. There is no differentiated structure of gynoecium and androecium when flower length is shorter than 1.5 mm, which was confirmed by microscopic anatomic dissection. We collected and preserved one single biological replicate from a tree individual. Because papaya male plants produce much more flowers than female trees do, we used 30 flowers from one male individual tree or 5 flowers from one female individual tree as a replicate, and then divided each sample into half (one for whole-genome bisulfite sequencing (WGBS) and the other for transcriptome sequencing). There were totally three biological replicates from three female individuals, as well as three replicates from three male individuals in each season. The volume of DNA and RNA extracted from all materials have basically met the minimum requirement for making libraries and sequencing.

We named female- and male- early-stage flower samples collected in spring, summer and winter as F, M, Fs, Ms, Fw and Mw, respectively. All samples were detected by multiplex RT-PCR and made sure that they are not infected by virus^14^.

### Whole-genome bisulfite sequencing (WGBS)

Whole-genome bisulfite sequencing library construction method was modified for low-quantity genomic DNA samples. We used 200 ng genomic DNA mixed with trace amounts of unmethylated λ DNA (*dam*-, *dcm*-) (Takara, Code No. 3019) to prepare WGBS library. The main steps are briefly described below: first, after DNA fragments of 300−500 bp were generated by sonication (Covaris Focused-ultrasonicator M220), we performed end-repair, dA-tiling and adaptor-ligation of DNA fragments using Ultra DNA Library Prep Kit (NEB, #E7370L) and multiplex methylated adaptor. Second, DNA with methylated adaptor was size-selected using beads (AxyGEN, lot.00517002) and bisulfite-treated using EpiTect Fast Bisulfite Kit (Qiagen, Lot.151043816) as per the product instructions. Third, the final bisulfite-conversed DNA with methylated adaptor was PCR-amplified using KAPA HIFI HotStart Uracil+ ReadyMix PCR kit (KAPA, KK2801) and index primers at 15 cycles to generate bisulfite-sequencing libraries. Finally, the indexed libraries were sequenced by Illumina HiSeq2500 platform to obtain 150-nt pair-end reads. Unfortunately, we lost some replicates due to their slight amounts of DNA. Because cold weather inhibits flowering of papaya in winter, we were not able to collect enough flowers in certain period of time as early-stage flowers were scarce. Therefore, flower samples in winter only have two biological replications.

### DNA methylation pattern and variance analysis

Trimmomatic script^15^ with default settings was used to remove low-quality bases or reads of raw data for generating clean reads. Using bismark aligner (version 0.15, http://www.bioinformatics.babraham.ac.uk/projects/bismark/), we aligned clean reads to papaya reference genome (Cpapaya_113_ASGPBv0.4, Phytozome). All resulting bam files were processed by R package “methykit”^16^ (version 1.2) to extract methylated Cs (cytosines) information from all libraries with at least six reads coverage and “merge” all samples to one object for base−pair locations that are covered in all samples (methylKit: User Guide) for further analysis: (1) Calculated methylation ratio (detected methylated Cs/total investigated Cs) for each cytosine locus. (2) The bisulfite conversion rate was evaluated from the average methylation ratio of exogenous unmethylated λ DNA. (3) Hierarchical cluster of the samples was plotted based on the Euclidean distance metric of percent methylation per base from 16 samples. (4) Differential methylation cytosines (DMCs) between every two sample groups were called using logistic regression by default parameters in “methylKit”. We also used analysis results of “methylKit” to extract the methylation ratio difference between each two sample groups. Only DMCs with a minimum difference of methylation ratio at 25% and *q* values < 0.01 were considered significant and chosen for downstream analysis. (5) We conducted genome-resequencing of pooled genomic DNA from all “Zhonghuang”-derived biological replicates. 25× sequencing depth of Illumina short reads was aligned by BWA^17^ and then calling SNPs by Freebayes^18^ (default parameters). Corresponding heterozygous SNPs of pooled samples were listed in Supplementary File 1. DMCs that showed same genomic position of heterozygous SNPs were filtered out to improve the credibility of comparative results.

We defined 2 Kb upstream regions of an annotated gene as its promoter sequence and 2 Kb downstream regions of an annotated gene as its downstream sequence. Hyper and hypo-methylated DMCs were the cytosine loci where methylations were observed at a higher and lower frequency than reference materials, respectively.

### Transcriptome profiling and differently expression gene (DEG) analysis

The same samples were also used for RNA-seq analysis. We extracted total RNA with Tripure reagent (Roche, Cat. No.11 667 165 001), and constructed RNA-seq libraries with Ultra RNA Library Prep Kit (NEB, #E7770L). Illumina HiSeq2500 system was used to perform the 100-nt or 150-nt pair-end reads high-throughput sequencing.

Trimmomatic^15^ script with default parameters was used to remove low-quality bases or reads of raw data to generate clean reads, which were then aligned to the papaya reference genome using Tohat2 aligner^19^, following instructions. Transcriptome reassembly and DEGs identification were carried out by Cufflinks 2.21^19^. DEGs with *p* values < 0.05 and *q* values < 0.01 were considered significant and chosen for downstream analyses. KEGG pathway enrichment analyses from well-known pathway databases were performed using KOBAS 3.0 online server^20,21^.

### Field observation of flowering time in male and female papaya plants

Seeds of papaya variety “Zhonghuang” were collected from fruits harvested in 2015 after hand pollination. We germinated the first batch of seeds from a single fruit on December 1, 2016, and the second batch of seeds from another fruit on December 15, 2016. All seedlings were planted in pots under 12-h day/night photoperiod, 25 °C, 55% relative humidity. Since the height of seedlings reached 35 cm 3 months later, we identified the sex type of seedlings using molecular markers and transplanted them in field (26.285°N, 118.487°E) on March 1, 2017. These papaya plants flowered in May 2017. To avoid subjective bias of measuring the flowering time, we counted the number of internodes when the first inflorescence emerged as a sign of beginning of flowering, considering the almost same rate of growth for all papaya seedlings. The lower number of internodes (counted from the ground) indicates the earlier flowering of plants.

### Statistical analysis and visualization of datasets

We used R (version 3.4.1) and TBtools Toolkit^22^ (version 0.664) to perform statistical analysis and visualization display. TBtools generated UpSet, heatmap plots and Venn diagram. Sankey diagram was produced by R package “ggalluvial”. DEGs were presented in the form of volcano diagram using R package “ggplot”. The visualization of average methylation ratios and corresponding methylome variances in SDR segments was displayed using R package “RIdeogram”. Pearson’s and Spearman’s correlation was calculated using R “cor” command.

## Results

### Genome-scale methylation profiling

To investigate the sex-specific methylome variances and dynamics in dioecious papaya, the early-stage flowers were bisulfite-sequenced across three seasons. The detailed information of bisulfite-sequencing data was provided in Supplementary Table 1, in which the sequencing depth of each library is more than 96×. The average methylation ratio of the exogenous λ DNA (unmethylated control) in each library was lower than 0.4%, which means that error rate caused by experiments was lower than 0.4% and the bisulfite conversion efficiency was higher than 99.6%. We chose the mC sites that were covered by at least six reads for analysis; the results showed that cytosines of three sequence sites (CpG, CHG and CHH, H stands for A, T or G) were methylated to different extent. There were 2,693,620 cytosines in CpG sequence, 3,045,577 in CHG, and 12,869,399 in CHH used for comparative analysis across 16 samples. We observed overall genome-scale average methylation ratio of 78.84−81.78%, 59.67−65.00% and 3.90−10.18% in CpG, CHG, and CHH, respectively (see Supplementary Table 2). Of these, the average cytosine methylation ratios of the spring male flowers were almost lowest, and the methylation ratios increased in the following summer and winter.

Based on the Euclidean distance metrics of methylation percent per base, 16 samples can be assigned to the specific clades by hierarchical clustering (see Supplementary Fig. 1), which suggested that the same sex type flowers grown in same season exhibit similar CpG and CHG methylome patterns. But it needs to be noted that, for CHH methylome, these samples of the same sex types could not be clustered in the same clades as that of CpG and CHG methylome did. The results suggested the more CHH methylation variations exist in the group of the same type flowers.

### Identification of sex-special methylation variation in comparisons between male and female flowers

**Fig. 1:**
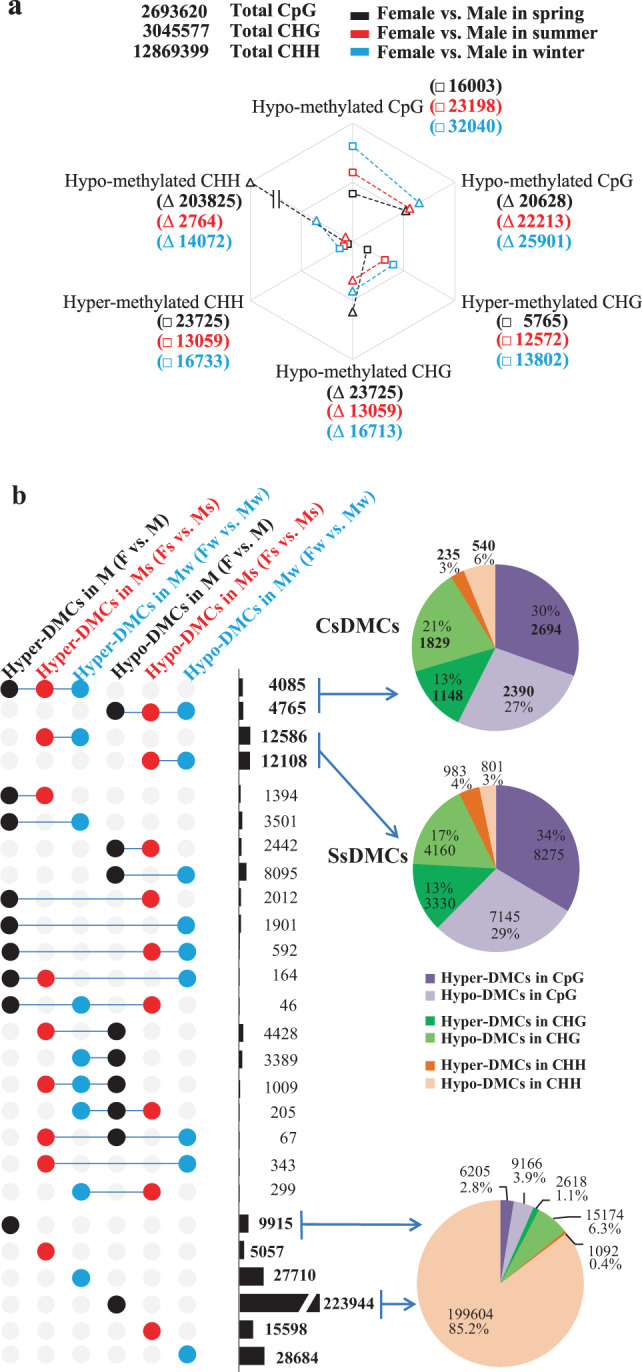
Statistics on the number of DMCs. **a** An overview of the male−female DMC amount in comparison with female and male flowers in three seasons (male vs. female control). The numbers in the brackets were the amounts of hyper-DMCs or hypo-DMCs in M, Ms or Mw when comparing their contemporary female flowers. **b** Intersections of male flowers DMCs. Left panel, colored circles linked with a horizontal line in each row represent the intersection of corresponding sets (hypo- or hyper-DMCs in M, Ms or Mw samples), and the number in right indicated shared number of DMCs in each intersection. The upper-right pie chart shows the distribution of CsDMCs in CpG, CHG and CHH contexts; the middle pie chart shows the distribution of SsDMCs; the chart in the lower right shows the distribution of novel DMCs in spring that was not detected in the other two seasons

The female flower samples grown in three different seasons (spring, summer and winter) were respectively used as the references to call the DMCs (differentially methylated cytosines) of male flower samples in the same seasons. Hyper-methylated DMCs (hyper-DMCs) were cytosine sites that have relatively high methylation, while hypo-methylated DMCs (hypo-DMCs) were sites that have relatively low methylation. By comparing with the spring female flowers, we observed the number of hypo-DMCs was far more than that of hyper-DMCs in spring male flowers, especially in the CHH context, which suggested that the spring male flowers showed low methylation pattern at most of the differentially methylated loci. However, for male flowers in summer or winter, the number of hypo-DMCs was nearly equal to that of hyper-DMCs (Fig. 1a).

Subsequently, we performed DMCs intersection among three seasons to investigate whether the methylation variations occur between male and female. UpSet plots (Fig. 1b) indicated that there were 4085 hyper-DMCs and 4765 hypo-DMCs constantly existing in male flowers of three seasons, compared to female flowers; thus those cytosine sites were regarded as conserved sex-special DMCs (CsDMCs). Among these DMCs, 2694 common hyper-methylated and 2390 common hypo-methylated were CpG context, accounting for more than half of total common CsDMCs. The number of CHH methylation variations was the least, which have accounted for less than one tenth of total common DMCs. In addition, we also found 12,586 common hyper-DMCs and 12,108 common hypo-DMCs only in both summer and winter male flowers when compared to female flowers, which should be regarded as stress-induced sex-special DMCs (SsDMCs). Similarly, the common DMCs in CpG context made up more than half of SsDMCs. The number of hyper-methylated and hypo-methylated SsDMCs was 8275 and 7145 in CpG contexts, 3330 and 4160 CHG contexts, 983 and 801 CHH contexts, respectively.

Despite CsDMCs and SsDMCs, majority of DMCs between male and female flowers varies. For instance, the 9915 hyper- and 22,394 hypo-DMCs (CHH-context DMCs were predominant) in comparison with spring male and female flowers did not arise again in the other two seasons. It revealed that the methylome was reprogrammed due to environmental changes.

### Most CsDMCs did not have a significant influence on neighboring genes regarding shaping their gene expression profiles

We hypothesized that the conserved CsDMCs in the genome might biologically affect the expression of neighboring genes; thus the transcriptional level of genes that contain CsDMCs was measured in order to study the influence of CsDMCs on neighboring genes. However, the transcription expression of 1216 protein-coding genes that have CsDMCs either in gene body or in 2 Kb upstream/downstream sequences showed that there was no similar trend in expression profiles between male and female flowers. We then focused on the genes that contain CsDMCs only in promoter regions. The correlation analysis indicated that the methylation ratio differences of CsDMCs in promoter regions between male and female flowers had no correlation with the expressional value changes of downstream coding genes (see Supplementary Table 3). The expression of these genes was inconsistent among the male and female flowers in different seasons. Both results indicated that the existence of most CsDMCs did not have an influence on expression of neighboring genes.

### Greater CpG and CHG methylation pattern similarity in female flowers than in male flower under stress

**Fig. 2:**
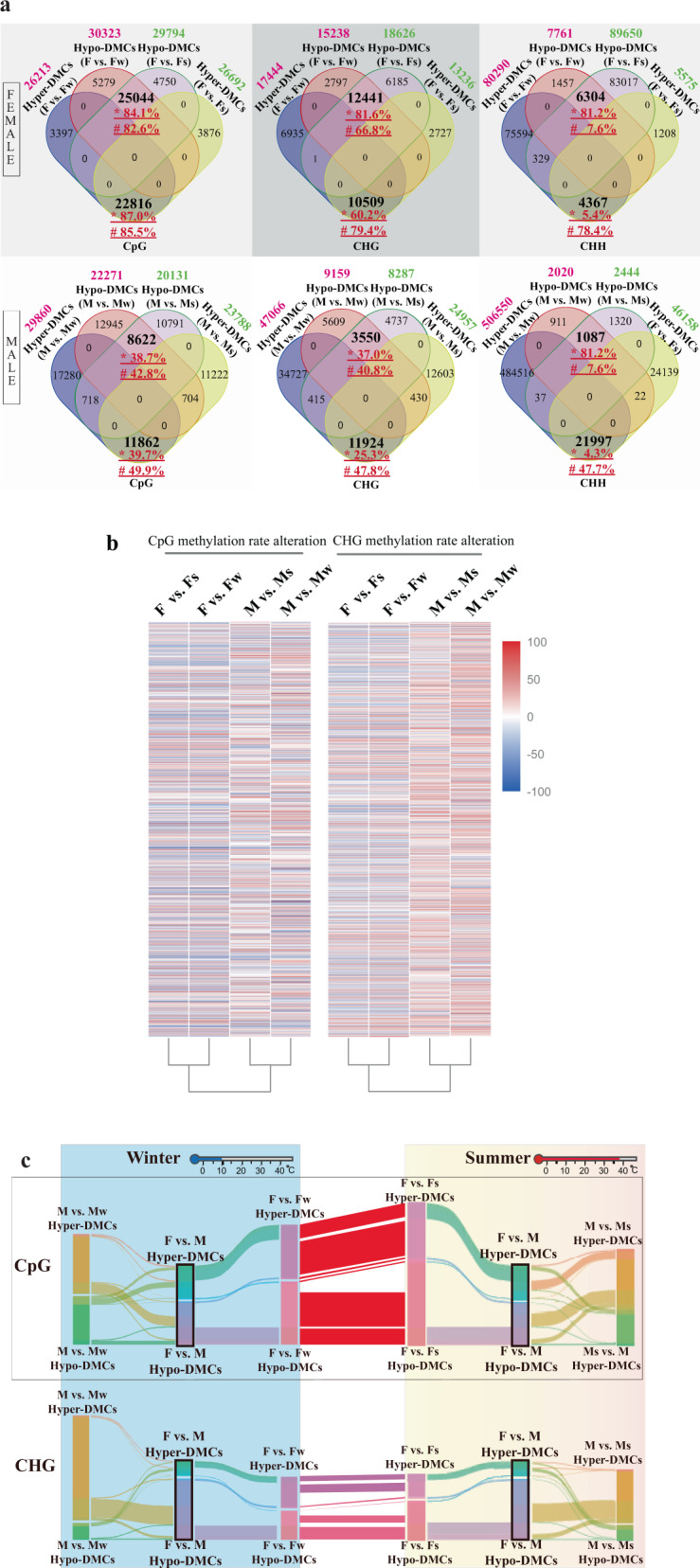
Methylation pattern changes in female and male flowers under stress. **a** Venn diagram of hyper-DMCs and hypo-DMCs in female and male flowers. The number in green shows the amount of hyper-DMCs or hypo-DMCs occurring in the flowers grown in summer; the number in magenta indicates the amount of hyper-DMCs or hypo-DMCs appearing in flowers grown in winter. Symbols * followed by the percentages represent the proportion of common hyper-DMCs or hypo-DMCs when comparing these common DMCs with hyper-DMCs or hypo-DMCs occurring in summer flowers; similarly, for #, the percentages were the proportion of common hyper-DMCs or hypo-DMCs when comparing them to hyper-DMCs or hypo-DMCs of winter flowers. **b** The methylation changes of female- and male-flowers DMCs in summer and winter. **c** Sankey diagram showing DMC changes in female and male flowers under different stress conditions. The DMCs with the same genomic locations were linked by streams across vertical blocks that represented hyper- or hypo-DMC sets in corresponding comparisons. The height of each vertical block represented the total number of relevant DMCs. The vertical height of stream line was inferred to the number of identical DMCs between the neighboring block. As seen in the figure, some blocks of DMC sets (F vs. M) could be clustered and linked to other sets, which means that these parts of DMCs occurring in female−male comparison have been reprogrammed in other types of flowers, that is, the methylation pattern of these cytosine loci was reset

When comparing the DNA methylome of both male and female flowers in spring with their counterparts in summer and winter, we found that a serial of differentiated methylation alterations responded to environmental stress in male and female flowers. Venn diagram (Fig. 2a) showed that more than 80% and 60% DMCs in CpG and CHG contexts, respectively, remained the same in female flowers under stress (grown in both summer and winter), while less than 50% of CpG-DMCs stayed the same in male flowers. CHH methylome provided an additional evidence of diverse methylation alteration in different sex type flowers under stress. In male flowers, hyper-DMCs outnumbered hypo-DMCs in both high- and low temperature, whereas for female flowers, the number of hyper-DMCs is greater in winter but hypo-DMCs outnumbered hyper-DMCs in summer.

We merged all CpG and CHG context DMC sites from four comparisons (F vs. Fs, F vs. Fw, M vs. Ms, M vs. Mw), to visualize the methylation alteration patterns. The degree of changes was evaluated by the methylation ratio difference between flower samples of summer/winter and spring flowers of the same sex (treated as control). Heatmap results of the changing degree revealed greater similarity of CpG and CHG methylation alteration in female flowers than that of male flowers, when they were grown under stress (Fig. 2b).

Furthermore, the DMCs analysis showed that the hyper-DMCs and hypo-DMCs in female and male flowers under stress condition are mostly different. It was found that a large number of DMCs between female and male flowers in spring were reprogrammed in male or female flowers grown in different seasons (Fig. 2c), which was the main reason that most male−female DMCs in spring could not be detected in the other two seasons.

### The expression profiling of genes involved in DNA methylation and chromatin structure regulation

**Fig. 3:**
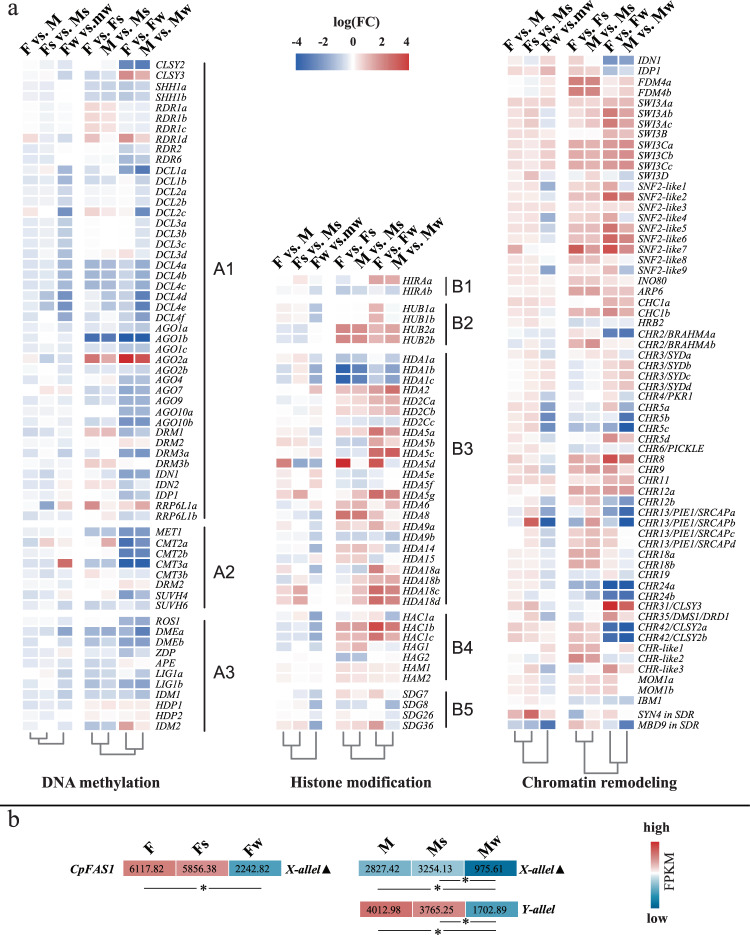
Methylation-related gene expression patterns changes in different comparisons. **a** Methylation-related gene expression changes. A1: genes involved in RNA-directed DNA methylation; A2: genes associated with DNA methylation maintenance; A3: genes associated with DNA demethylation; B1: genes belong to *Histone Chaperone HIRA*; B2: genes similar to *HISTONE MONOUBIQUITINATION1*; B3: *Histone deacetylase*; B4: *Histone acetyltransferase*; B5: *SET-domain group genes*. For corresponding gene IDs, see Supplementary Table 4. **b** Gene expression of *CpFAS1* alleles in SDR. Filled triangle: this allele was predicted as pseudogene^9,25^. Asterisk designated a significant difference (*P* < 0.05) between two sample groups by DEG analyses

The dynamic regulation in DNA methylation included the establishment, maintenance and demethylation of 5-mC ^23^. The methylation state was also highly associated with the adjustment of chromatin structure which was dependent on the histone modification and chromatin remodeling^24^. Thus, we performed the expression profiling of the related genes involved in DNA methylation, histone modification and chromatin remodeling to analyze whether these changes have affected the DNA methylation status in different samples to confer the appropriate adaptation response of developmental stimuli and environmental stresses (Fig. 3a). When comparing the male flowers and their female counterparts (F vs. M, Fs vs. Ms, Fw vs. Mw) in different seasons, the results showed that the genes related to DNA methylation, histone modification or chromatin remodeling exhibit distinct expression patterns among different seasons, which was one of the reasons that the conserved DMCs were fewer when comparing methylome between male and female flowers. Beyond that, when comparing the flowers in summer or winter against the ones collected in spring, we observed that, in the same seasons, male and female flowers displayed similar gene expression changes, which means the result that previously mentioned greater CpG and CHG methylation pattern similarity of female flowers than male flowers under stress, should be caused by the smaller-scale differences in mRNA levels of one or some key genes, rather than a whole range of genes participating in the DNA methylation, histone modification, or chromatin-remodeling processes.

The difference of genetic background between male and female plants of papaya variety “Zhonghuang” was mainly defined in the SDR section. So we searched for the DEGs of the same type flowers grown in summer and winter, and found a chromatin-remodeling-related *CpFAS1* (the ortholog of *FASCIATA1/ FUGU2*) had significantly decreased its transcription expression in winter (Fig. 3b). *CpFAS1* located in SDR was annotated as a functionally Y-chromosome-special gene since its X chromosome counterpart gene was regarded as a pseudogene^12,23^.

### Sex-associated DEGs underlined the phytohormone signal transduction and had no apparent association with sex-special DMCs

**Fig. 4:**
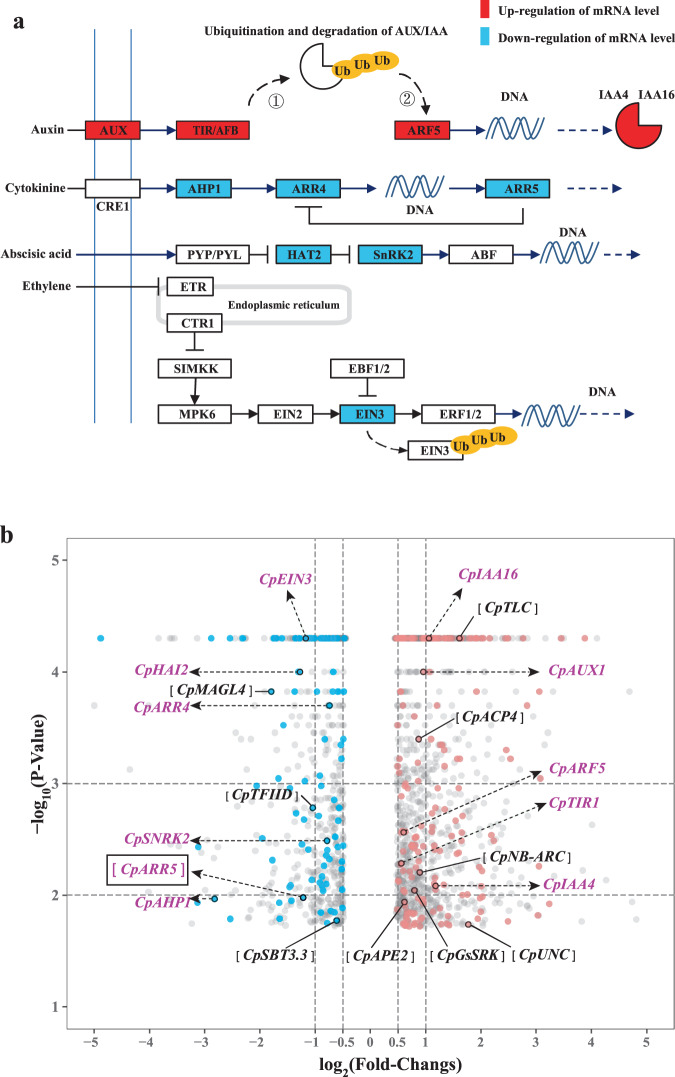
DEGs analyses and visualizations in corresponding comparisons. **a** KEGG enrichment analysis of sex-associated DEGs underlines the phytohormone signal transduction in male flowers when compared to female ones. Auxin-related TIR1/AFBs signaling transduction: auxin was transported by membrane-bound carrier AUX (*CpAUX1* encoding) and bound to receptor TIR/AFBs (*CpTIR1* encoding). ① Complex containing TIR/AFBs triggered subsequently ubiquitin-mediated proteolysis of AUX/IAA by recruiting proteasome; ② Aux/IAA degradation alleviates the transcript repression of ARF5 gene *CpARF5*. In the last step, ARF5 bound the DNA elements and promoted the transcription of IAA gene (*CpIAA4* and *CpIAA6*). Ub in yellow circles indicate the degradation of target proteins via the ubiquitin proteasome pathway. **b** Volcano diagram showing the DEGs in spring male flowers when compared with the spring female counterparts. Each dot represents individual DEG, the red and blue dots indicate upregulated and downregulated sex-associated DEGs in male flowers, respectively; gray dots represent DEGs excluding sex-associated DEGs. Plotted −log_10_ (*P* value of DEGs) on the vertical *y*-axis against log_2_ (fold-changes of DEGs) on the horizontal *x*-axis. Eleven sex-associated DEGs involved in phytohormone signal transduction were marked as green labels, and nine sex-associated DEGs with identical methylation variances were marked as black labels. *CpARR5* is earmarked by rectangle as the only phytohormone signal-responsive DEG associated with a hypomethylated methylation variance. Nine other DEGs were *CpACP4* (encoding acyl carrier protein 4, two CsDMC in third introns and three CsDMCs in downstream region), *CpAPE2* (encoding chloroplast triose phosphate/3-phosphoglycerate translocator, five CsDMCs in promoter)*, CpGsSRK* (encoding G-type lectin S-receptor-like serine/threonine-protein kinase, one CsDMCs in downstream), *CpMAGL4* (encoding alpha/beta-Hydrolases superfamily protein, four CsDMCs in third introns and three CsDMCs in downstream), *Cp NB-ARC* (encoding NB-ARC domain-containing disease resistance protein, three CsDMCs in promoter), *CpSBT3.3* (encoding Subtilase family protein, one CsDMCs in fourth exon), *CpTFIID* (encoding Transcription initiation factor TFIID subunit 8, one CsDMCs in first exon), *CpTLC* (encoding TRAM, LAG1 and CLN8 lipid-sensing domain-containing protein, one CsDMCs in promoter and one CsDMCs in downstream), *CpUNC* (encoding uncharacterized conserved protein, one CsDMCs in downstream). For related CsDMCs genomic positions, see Supplementary Table 6

Comparing male and female flowers in three seasons respectively (M vs. F, Ms vs. Fs, Mw vs. Fw), there were 243 common DEGs between two sexes across three seasons, which were considered to be sex-associated DEGs (197 genes were upregulated and 104 downregulated in male flowers when compared to female ones). Based on KEGG metabolic pathways enrichment analysis of sex-associated DEGs, with corrected *P* value < 0.0001, phytohormone signal transduction was the only significantly enriched metabolic pathway. Eleven sex-associated DEGs were annotated in phytohormone signal transduction, including *CpAUX1, CpTIR1*, *CpARF5*, *CpIAA4*, *CpIAA16*, *CpAHP1*, *CpARR4*, *CpARR5*, *CpSnRK2*, *CpHAI2*, *CpEIN3* (for gene IDs and expression levels, see Supplementary Table 5). Of these DEGs, the genes involved in auxin-related TIR1/AFBs signaling transduction, consisting of *CpAUX1, CpTIR1*, *CpARF5*, *CpIAA4* and *CpIAA16*, showed an ascending trends in mRNA level (Fig. 4a). Enhanced TIR1/AFBs signaling transduction was detected in male flowers compared to female flowers among the three seasons.

It is not clear about the relation between sex-associated DEGs and CsDMCs. According to positional mapping of sex-associated DEGs and CsDMCs in the genome, distinct genomic locations between them suggested that most sex-associated DEGs probably did not have an association with sex-associated DMCs. However, at least 10 out of 243 genes could be exceptions. Among these ten sex-associated DEGs, *CpARR5*, with the decrease in gene transcription expression in male flowers, was the only one phytohormone signal-responsive sex-associated DEG harboring a hypo-methylated CHH locus in downstream region. Besides, nine sex-associated DEGs, including *CpACP4*, *CpAPE2*, *CpGsSRK*, *CpMAGL4*, *CpNB-ARC*, *CpSBT3.3, CpTFIID, CpTLC*, and a putative gene had CsDMCs within the gene body or flanking sequences (Fig. 4b).

### DNA methylation profiling in SDR section

**Fig. 5:**
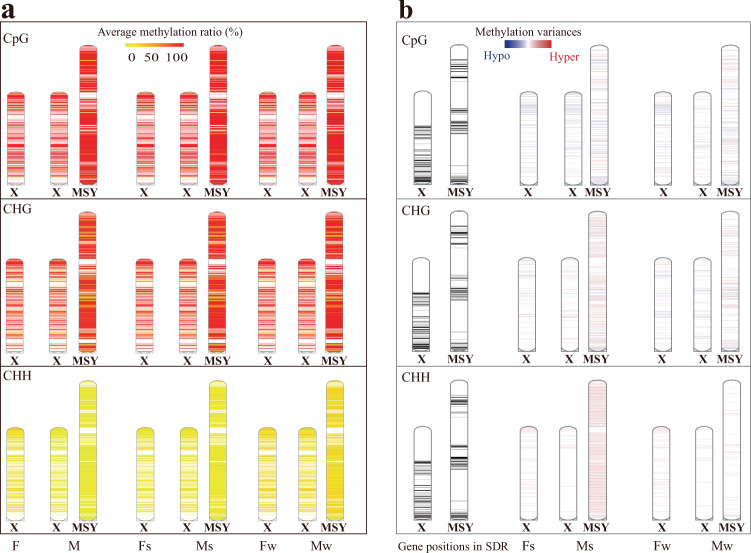
The methylation pattern of SDR. **a** The average methylation ratio of SDR (1000 bp bin tiling). **b** DMC (methylation variances) distribution of flowers grown in summer and winter in SDR. DMC distributions were shown where methylation of either summer or winter flowers was significantly different from that of spring flowers, respectively. The chromosomal diagram on the left showed the position of genes in SDR (gene positions were represented by black blocks), while diagrams in the middle and on the right indicated DMC positions in SDR (relative positions of DMCs indicated by horizontal lines with different colors, which ranged from red to blue representing the methylation ratio difference was from +100% to −100%)

The sex of papaya was determined by genes in the sex determination region (SDR) of nascent sex chromosomes. To access DNA methylation patterns of the SDR, we investigated the methylation status of SDR and its X counterpart in different flowers samples (Fig. 5a). Using 1000 bp bin tiling strategy, we calculated the average methylation ratio and found that CpG and CHG methylation ratios were high in most SDR, that is, SDR was heavily methylated in CpG and CHG contexts. CHH context showed low methylation compared to CpG and CHG. Only when the flowers were exposed under low temperature in winter, some segments of SDR were hyper-methylated at CHH loci.

SDR-wide DMC profiling in different types of flowers showed that the overall X and SDR regions in male flowers had more and wider-range methylation variances than X counterparts in female flowers did (Fig. 5b).

### Early flowering of papaya “Zhonghuang” male plants may be associated with the variations of transcript-level expression of autosomal *SVP* and *APETALA1* under epigenetic control

**Fig. 6:**
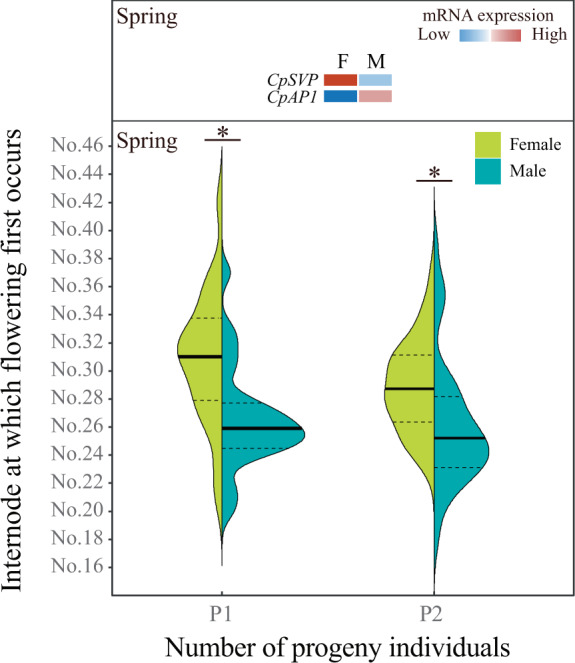
Field observation of flowering time in male and female progeny. The violin plot showed the continuous numeric data distribution of the internode position at which first inflorescence occurred in female and male plants. The inner part of the violin plot shows the mean (heavy line) and the interquartile range (dotted line). P1: the first batch of seedlings germinated on December 1, 2016; P2: the second batch of seedlings germinated on December 15, 2016. Asterisk designated a significant difference (*t* test, *P* < 0.05) of inflorescence initiation between male and female progeny

The average methylation ratios of the male flowers in spring were the lowest, which aroused great interest in investigating the possible consequences of genome-wide hypomethylation in male trees. When comparing male flowers (spring) to female flowers (spring), we have noticed the presence of three hypo-DMCs (two in first intron, one in second exon) of *CpSVP* (encoding SHORT VEGETATIVE PHASE, evm.TU.supercontig_55.31), which is an essential antagonistic flowering regulator, as well as its significantly decreased mRNA level. Both evidences suggested that this gene has possibly been regulated by epigenetic processes. *CpSVP* gene expression under possible epigenetic repression potentially affected flowering time. The field statistical analyses in spring showed there are less internodes (lower height) when inflorescences of male trees initiate, compared with that of female counterparts, which indicated that male progeny of papaya “Zhonghuang” exhibits naturally occurring early flowering (Fig. 6). The epigenetic regulation of flowering time could also be supported by another piece of evidence from *APETALA1* (*AP1*). Since *AP1* is considered as a crucial regulator in floral meristem identity through abrogating downstream floral repressor, we performed a comparison of *CpAP1* transcript expression and its methylation status between male and female flowers. The mRNA level of *CpAP1* (evm.TU.supercontig 1.162) in male flower increased and two low-frequency methylated CHH loci were found in its promoter.

## Discussion

### Interrelation between sex-special methylation variances and differential expression genes as well as the possible function of *cpARR5*

Genome-wide bisulfite sequencing (WGBS) was performed to investigate the sex-special DNA methylation in papaya male and female flowers. Nearly 9000 CsDMCs were stably detected in different sexual flowers across three seasons. Those conserved CsDMCs among different seasons should be a minimal evidence of DNA methylation variances between female and male papaya flowers. The sex-special methylation variances might contribute to the formation of sex characteristics by mediating the gene expression. However, we found that most nearby genes harboring CsDMCs did not show the similar change patterns in the transcriptional level when comparing male and female flowers in three different seasons. The biological significance of these epigenetic differences were still unclear, considering most CsDMCs either in or near coding gene regions had no broad influences on the related gene expression. Using targeted analysis, we have identified ten sex-associated DEGs that flanked sequences or gene bodies containing CsDMCs. Out of these genes, *CpARR5* was the only phytohormone-signaling-responsive sex-associated DEG harboring CsDMC. Interestingly, in our previous transcriptional analysis, the functional and rudimentary gynoecium of different sexual papaya flowers show the most significant difference in phytohormone signal transduction^26^. In the present study, phytohormone signaling was also considered relevant to papaya sexual differences. Based on the results of these two studies, phytohormone homeostasis alteration might profoundly influence the characteristics in different sex types of papaya. If so, a potentially epigenetic targeting of *CpARR5* might mediate papaya inflorescence development, considering that *CpARR5* was found to be the only phytohormone signal-responsive DEG associated with an obvious methylation variance between the two opposite sexes (see Results, Fig. 4).

ARR5 was a crucial type-A ARR transcription repressor in cytokinin signaling^27^. In *Arabidopsis* mutants, the defect of cytokinin signaling-related ARR gene family can elongate petiole length, inhibit roots elongation and lateral root formation. Some evidences support that cytokinin signaling could crosstalk with floral MADS-box proteins to affect inflorescence architecture^28^. Disruption of cytokinin homeostasis could diminish gynoecium cell proliferation and have a significant influence on the morphology of floral organ^29,30^. *AtARR16* (a paralog of *AtARR5*) accompanied with *AtAHP6* (encoding Histidine Phosphotransferase 6) in *Arabidopsis* has been reported to control the cytokinin signaling and participate in the growth of young gynoecium^31^. *PbRR9*, an ortholog of *AtARR16* at Y chromosome, was thought to be the sex-determining gene with a marked differential methylated promoter and the first intron being epigenetically mediated in *Populus balsamifera*^32^. Cytokinin and ABA signaling always antagonistically control plant organ development and stress response, and this process could be implemented though increasing protein stability of ARR5, which was phosphorylated by SnRK2 of ABA signaling^33^. In our study, mRNA transcription levels of both epistatic *CpSnRK2* and downstream *CpARR5* were lower in male flowers than that of female flowers (see Fig. 4a), which implied that the interplay of cytokinin and ABA signaling for development might be different in male and female flowers. It was possible for male flowers that disturbance of cytokinin signaling in floral organ primordia had severely interfered in the morphogenesis of early gynoecium and resulting in residual tissue of female sterility. The decrease of *CpARR5* expression level might be the result of inflorescence differentiation and have nothing to do with the gynoecium and androecium formation. However, the expression of type-A *CpARR* show similar decreasing patterns in male flowers and their rudimentary gynoecium (comparing to female flowers or their functional gynoecium), which needs further investigation of whether a developmental cascade could be triggered by *CpARR5* and then lead to sexual differentiation.

### Diverse stress-responsive methylations in male and female flowers

The stress-responsive methylomes were of a slight difference in papaya flowers under extreme temperatures. The female flowers exhibited similar methylome alteration, while the male flowers displayed more malleable methylome changes when comparing them growing in hot and cold temperature. Interestingly, similar methylation alteration occurs in SDR region in male flowers and its counterpart in female flowers. Methylome variability in SDR of male flowers might result in epigenetic control of floral sex differentiation under stress conditions, through switching key sex determinant on and off.

Comparing the summer and winter flowers with the spring flowers, respectively, we found that the transcriptional expression change patterns of methylation-related genes were similar, regardless of male or female flowers, when they were exposed to the same adverse climatic conditions. This finding rules out a possibility that the different methylation patterns of male flowers between summer and winter were caused by seasonally differential changes of methylation-related genes. Because of the same climate and similar gene changes, the methylome variances of female flowers between summer and winter were less than those of male flowers. Thus, the more stress-responsive methylation variances that were vulnerable to climate changes in male flowers might be attributed to some specific genes or lack of specific genes in their gene-poor MSY region (MSY is the only genomic difference in male papaya compared to female papaya).

In comparison with the gene expression in MSY and its X counterpart between summer and winter male flowers, differentially expressed chromatin-remodeling-related *CpFAS1* was contracted. MSY-specific *CpFAS1* (its X counterpart is pseudogene^9,25^) was downregulated in winter male flowers, while its expression in spring and summer was nearly unchanged. FAS1 was identified as a subunit of Chromatin Assembly Factor 1 (CAF1). In *Arabidopsis* mutant, chromatin conformation was severely affected by very low mRNA expression level of *AtFAS1*, causing reduced heterochromatin content, as well as loss of telomere and 45S rDNA^34–37^. Additionally, *FAS1* was reported to participate in auxin-mediated TIR1/AFBs signaling-responsive chromatin accessibility pathway^38^. From comparative transcriptome analysis, we found an enhanced TIR1/AFBs signaling transduction in male flowers compared to female flowers in all three seasons. Both findings about the male-specific *CpFAS1* in MSY and the changes of TIR1/AFBs signaling transduction implied that the chromatin accessibility of male flowers might be different from that of female flowers. Therefore, when male flowers grow under different temperature stress, the larger adjustment of chromatin conformation caused by male-specific *cpFAS1* and TIR1/AFBs signaling would alternatively affect the facilitated access of methylation-related enzymes or restricted their recruitment for assembling a functional chromatin assembly complex, which might cause variable methylomes of male flowers. In contrast, the absence of large-scale alterations in chromatin accessibility in female flowers led to stable stress-responsive methylome and showed similar methylome patterns.

### *CpSVP* and *CpAP1* gene-specific intergenic hypomethylation may contribute to the early flowering of male papaya in spring

The transition to flowering is one of the major phase changes during plant life cycle, which is mediated (activated or repressed) by the expression of a variety of genes and is susceptible to various environmental factors such as temperature and photoperiod. In our study, it was observed that the related sequences of MADS transcription factors genes *CpSVP* and *CpAP1* contained some DMCs.

Flowering repressor *SVP* and flowering activator *AP1* can affect flowering time in plants^39,40^. As a flowering repressor, *AtSVP* suppresses precocious flowering in *Arabidopsis* under both long and short days^41^; overexpressing *EgrSVP* in *Brassica* plants exhibited late flowering^42^; overexpressing *AcSVP1* and *AcSVP4* in kiwifruit could delay dormancy release and flowering^43^. In contrast, *AP1* promotes flowering^40,44^. The phenotype of transgenic plants and mutants indicated that *VRN1* (*AP1* homolog in wheat) is a flowering activator that had an indispensable role in the floral transition of wheat^45^. Heterologous expression of *GmAP1* derived from soybean caused early flowering and floral organs alteration in tobacco^46^. Overexpression of longan *DlAP1* genes in transgenic *Arabidopsis* resulted in a significantly early flowering phenotype^47^. Considering these evidences, with the combined analysis of the gene expression data in papaya male and female flowers in spring season, we thought that *CpSVP* and *CpAP1* can also participate in the transition from vegetative to reproductive growth, and have an important influence on flowering time. Our data showed that the male papaya with differential downward stream of *CpSVP* transcript had flowered much earlier than female papaya which had higher expression of *CpSVP*. It indicated that, in male papaya, early flowering may be partly related to the decreased expression of the floral transition inhibitor *CpSVP*. On the other hand, increasing the expression of *CpAP1* could initiate and accelerate flowering of male papaya after the hypermethylation suppression of the *CpAP1* promoter was removed, based on the phenomenon of two hypomethylated CHH loci in the *CpAP1* promoter of male flowers. We proposed that in male trees activating *CpAP1* and suppressing *CpSVP* initiates floral development earlier, where gene expression of both genes could be regulated by methylation to some degree.

DNA methylation state has already been proved to have the ability to affect flowering time. In an apomictic clonal lineage of *Taraxacum officinale*, heritable differences of flowering time were found to be the consequence of the DNA methylation variation^48^. Transiently disrupting DNA methylation of the floral promoter induced late flowering behavior in bread wheat^49^. Using the DNA methyltransferase inhibitor Zebularine or 5-Azacytidine, flowering-related genes were activated by DNA demethylation in favor of earlier flowering in *Pharbitis nil*^50^ and *Linum usitatissimum*^51^. Similarly, some flowering initiating genes (such as *CiAP1*, *CiFLC*, and *CiLFY*) were influenced by locus-specific methylation alteration with 5-azacytidine hypomethylation treatment, and might play an important role in early flowering and juvenescent phase shortening in *Poncirus trifoliata*^52^. However, several deleterious effects of DNA demethylating agent have also been reported as dwarfism or other weak growth performance^50,53^. In our unreported work, similar methylation inhibition experiment was conducted. 20 and 40 μM Zebularine or 5-Azacytidine was sprayed towards inflorescences on the junction of stem and leaf petiole near apex of the trunk. We used the same trees (three male biological replications and three female biological replications that were chosen for methylome and transcriptome analyses) to study methylation inhibitory effects with Zebularine or 5-Azacytidine spraying once every 2 weeks for 4 weeks. However, we found that papaya was vulnerable to demethylation agent. Demethylating agent application did not have striking changes on morphological characteristic of inflorescences during the first week. From the second week, all papaya trees began to show small and deeply pinnate lobed leaves, along with the senescence of newly appeared flower buds. Drug-induced demethylation effects made papaya trees show severe inhibition of inflorescences growth in both male and female plants. Similar growth inhibitory effects were reported in *Arabidopsis* and other plant species with demethylation agent treatment^53,54^. Severe growth inhibitory effects with withered young leaves were observed in young papaya seedlings as well.

### The evolutionary benefits of sex-specific methylome variances in male papaya

Methylome variability of MSY in male flowers might have the attributes of epigenetic control in seasonal appearance of deformed floral development^55^ and sex reversal in papaya^11,12^. In comparison with female, male papaya showed more phenotypic plasticity in adapting to environmental changes. After a cold winter, the recovery of female fertility frequently occurs in some male flowers^56^. These male-to-hermaphrodite sex reversal flowers can produce germinable seeds to increase individuals in wild population of dioecious papaya. On the other hand, within a new population generated by seeds, young male papaya seedlings might gain the ability of early flowering from hypomethylation and transcriptional inactivation/activation of specific flowering-related genes in spring season. As a result, early-flowering male plants will further ensure reproductive success when females are mature.

Previous studies have proposed the epigenetic influence on sex determination as well as sex-chromosome evolution. In genetically determined species, *Carica papaya*, DNA hypermethylation and heterochromatinization of sex-specific nonrecombining region was the distinguishing character of primitive Y chromosome. For *Silene latifolia* with heteromorphic XY sex chromosome, it was reported that Y allelic genes in older stratum were more methylated and transposable element accumulation may cause the heavy DNA methylation to coincide with heterochromatinization in physically larger Y chromosome^57^. The distribution of histone modification biomarkers demonstrated contrasting active histone modification pattern between heteromorphic XY chromosome, which revealed the possible genetic imprint in sex chromosomes of dioecious plants^58^. These recent findings linked DNA methylation and histone covalent modifications with sex-chromosome evolution. Our analyses of the inter-seasonal methylation variations of dioecious flowers will further test the hypothesis of DNA methylation and heterochromatinization in nonrecombining region of the nascent sex chromosomes in papaya.

## Supplementary information

Revised_manuscript_Supplementary_Figure 1.pdf

Revised_manuscript_Supplementary_Figure 2.pdf

Revised_manuscript_Supplementary_Table 1.pdf

Revised_manuscript_Supplementary_Table 2.pdf

Revised_manuscript_Supplementary_Table 3.pdf

Revised_manuscript_Supplementary_Table 4.pdf

Revised_manuscript_Supplementary_Table 5.pdf

Revised_manuscript_Supplementary_Table 6.pdf

Revised_manuscript_Supplementary_file 1.rar

## Data Availability

All high-throughput sequencing data of methylome and transcriptome reported in this paper were publicly accessible in Genome Sequence Archive (GSA, https://bigd.big.ac.cn/gsa) in BIG Data Center (Beijing Institute of Genomics, Chinese Academy of Sciences) under GSA accession numbers CRA002034, CRA001995.

## References

[1] Ming R, Bendahmane A, Renner SS (2011). Sex chromosomes in land plants. Annu. Rev. Plant Biol..

[2] Aryal R, Ming R (2014). Sex determination in flowering plants: papaya as a model system. Plant Sci..

[3] Juarez C, Banks JA (1998). Sex determination in plants. Curr. Top. Dev. Biol..

[4] Akagi T, Henry IM, Kawai T, Comai L, Tao R (2016). Epigenetic regulation of the sex determination gene MeGI in polyploid persimmon. Plant Cell.

[5] Akagi T, Henry IM, Tao R, Comai L (2014). A Y-chromosome-encoded small RNA acts as a sex determinant in persimmons. Science.

[6] Janousek B, Siroký J, Vyskot B (1996). Epigenetic control of sexual phenotype in a dioecious plant, Melandrium album. Mol. Gen. Genet.

[7] Martin A (2009). A transposon-induced epigenetic change leads to sex determination in melon. Nature.

[8] Liu Z (2004). A primitive Y chromosome in papaya marks incipient sex chromosome evolution. Nature.

[9] Vanburen R (2015). Origin and domestication of papaya Yh chromosome. Genome Res..

[10] Yu Q (2008). Recent origin of dioecious and gynodioecious Y chromosomes in papaya. Trop. Plant Biol..

[11] Lin H, Liao Z, Zhang L, Yu Q (2016). Transcriptome analysis of the male-to-hermaphrodite sex reversal induced by low temperature in papaya. Tree Genet. Genomes.

[12] Hofmeyr JDJ (1939). Sex reversal in carica Papaya L. South Afr. J. Sci..

[13] Zhang W, Wang X, Yu Q, Ming R, Jiang J (2008). DNA methylation and heterochromatinization in the male-specific region of the primitive Y chromosome of papaya. Genome Res..

[14] Tuo D (2014). Development and validation of a multiplex reverse transcription PCR assay for simultaneous detection of three papaya viruses. Viruses.

[15] Bolger AM, Lohse M, Usadel B (2014). Trimmomatic: a flexible trimmer for Illumina sequence data. Bioinformatics.

[16] Akalin A (2012). methylKit: a comprehensive R package for the analysis of genome-wide DNA methylation profiles. Genome Biol..

[17] Li H, Durbin R (2010). Fast and accurate long-read alignment with Burrows-Wheeler transform. Bioinformatics.

[18] Garrison, E. & Marth, G. Haplotype-based variant detection from short-read sequencing. *arXiv* **1207**, 3907 (2012).

[19] Trapnell C (2012). Differential gene and transcript expression analysis of RNA-seq experiments with TopHat and Cufflinks. Nat. Protoc..

[20] Wu J, Mao X, Cai T, Luo J, Wei L (2006). KOBAS server: a web-based platform for automated annotation and pathway identification. Nucleic Acids Res..

[21] Xie C (2011). KOBAS 2.0: a web server for annotation and identification of enriched pathways and diseases. Nucleic Acids Res..

[22] Chen, C., Xia, R., Chen, H. & He, Y. TBtools, a Toolkit for biologists integrating various HTS-data handling tools with a user-friendly interface. *bioRxiv.*10.1101/289660 (2018).

[23] Elhamamsy AR (2016). DNA methylation dynamics in plants and mammals: overview of regulation and dysregulation. Cell Biochem. Funct..

[24] Zhang H, Lang Z, Zhu J-K (2018). Dynamics and function of DNA methylation in plants. Nat. Rev. Mol. Cell Biol..

[25] Wang J (2012). Sequencing papaya X and Yh chromosomes reveals molecular basis of incipient sex chromosome evolution. Proc. Natl. Acad. Sci. USA.

[26] Zhou P, Fatima M, Ma X, Liu J, Ming R (2019). Auxin regulation involved in gynoecium morphogenesis of papaya flowers. Hortic. Res..

[27] Eva H, Nijuscha G, Alexander H (2010). The more, the merrier: cytokinin signaling beyond Arabidopsis. Plant Signal Behav..

[28] Han Y, Yang H, Jiao Y (2014). Regulation of inflorescence architecture by cytokinins. Front. Plant Sci..

[29] Nayelli MM (2012). The role of cytokinin during Arabidopsis gynoecia and fruit morphogenesis and patterning. Plant J. Cell Mol. Biol..

[30] Isabel B, Elisabeth O, Miroslav S, Tomáš W, Thomas S (2011). Cytokinin regulates the activity of reproductive meristems, flower organ size, ovule formation, and thus seed yield in *Arabidopsis thaliana*. Plant Cell.

[31] Reyes-Olalde JI (2017). The bHLH transcription factor SPATULA enables cytokinin signaling, and both activate auxin biosynthesis and transport genes at the medial domain of the gynoecium. PLoS Genet..

[32] Bräutigam K (2017). Sexual epigenetics: gender-specific methylation of a gene in the sex determining region of Populus balsamifera. Sci. Rep..

[33] Huang X (2018). The antagonistic action of abscisic acid and cytokinin signaling mediates drought stress response in Arabidopsis. Mol. Plant.

[34] Varas, J., Santos, J. L. & Pradillo, M. The absence of the Arabidopsis chaperone complex CAF-1 produces mitotic chromosome abnormalities and changes in the expression profiles of genes involved in DNA repair. *Front. Plant Sci.* **8**, 525 (2017). 10.3389/fpls.2017.00525 PMC5386969 28443118

[35] Pavlištová Veronika, Dvořáčková Martina, Jež Michal, Mozgová Iva, Mokroš Petr, Fajkus Jiří (2016). Phenotypic reversion infasmutants ofArabidopsis thalianaby reintroduction ofFASgenes: variable recovery of telomeres with major spatial rearrangements and transcriptional reprogramming of 45S rDNA genes. The Plant Journal.

[36] Veronika M (2015). Homology-dependent repair is involved in 45 S rDNA loss in plant CAF-1 mutants. Plant J. Cell Mol. Biol..

[37] Iva M, Petr M, Jirí F (2010). Dysfunction of chromatin assembly factor 1 induces shortening of telomeres and loss of 45 S rDNA in Arabidopsis thaliana. Plant Cell.

[38] Hasegawa J (2018). Auxin decreases chromatin accessibility through the TIR1/AFBs auxin signaling pathway in proliferative cells. Sci. Rep..

[39] Aerts N, Bruijn SD, Mourik HV, Angenent GC, Dijk ADJV (2018). Comparative analysis of binding patterns of MADS-domain proteins in Arabidopsis thaliana. Bmc Plant Biol..

[40] Kerstin K (2010). Orchestration of floral initiation by APETALA1. Science.

[41] Hartmann U (2010). Molecular cloning of SVP: a negative regulator of the floral transition in Arabidopsis. Plant J..

[42] Lee JH, Park SH, Lee JS, Ji HA (2007). A conserved role of SHORT VEGETATIVE PHASE (SVP) in controlling flowering time of Brassica plants. Biochim. Biophys. Acta Gene Struct. Expr..

[43] Wu R, Wang T, Allan AC, Macknight RC, Varkonyi-Gasic E (2018). Overexpression of both AcSVP1 and AcSVP4 delays budbreak in kiwifruit A. chinensis var. deliciosa, but only AcSVP1 delays flowering in model plants. Environ. Exp. Bot..

[44] Mandel MA, Yanofsky MF (1995). A gene triggering flower formation in Arabidopsis. Nature.

[45] Sanae S (2009). A genetic network of flowering-time genes in wheat leaves, in which an APETALA1/FRUITFULL-like gene, VRN1, is upstream of FLOWERING LOCUS T. Plant J..

[46] Chi Y, Fang H, Liu H, Yang S, Yu D (2011). An APETALA1 -like gene of soybean regulates flowering time and specifies floral organs. J. Plant Physiol..

[47] Winterhagen P, Tiyayon P, Samach A, Hegele M, Wünsche JN (2013). Isolation and characterization of FLOWERING LOCUS T subforms and APETALA1 of the subtropical fruit tree Dimocarpus longan. Plant Physiol. Biochem..

[48] Wilschut RA, Oplaat C, Snoek LB, Kirschner J, Verhoeven KJF (2016). Natural epigenetic variation contributes to heritable flowering divergence in a widespread asexual dandelion lineage. Mol. Ecol..

[49] Finnegan EJ (2018). Zebularine treatment is associated with deletion of FT-B1 leading to an increase in spikelet number in bread wheat. Plant, Cell Environ..

[50] Iwase Y, Shiraya T, Takeno K (2010). Flowering and dwarfism induced by DNA demethylation in Pharbitis nil. Physiologia Plant..

[51] Brown JCL, De Decker MM, Fieldes MA (2008). A comparative analysis of developmental profiles for DNA methylation in 5-azacytidine-induced early-flowering flax lines and their control. Plant Sci..

[52] Zhang Jin-Zhi, Mei Li, Liu Rong, Khan Muhammad Rehman Gul, Hu Chun-Gen (2014). Possible Involvement of Locus-Specific Methylation on Expression Regulation of LEAFY Homologous Gene (CiLFY) during Precocious Trifoliate Orange Phase Change Process. PLoS ONE.

[53] Herden J, Eckert S, Stift M, Joshi J, van Kleunen M (2019). No evidence for local adaptation and an epigenetic underpinning in native and non-native ruderal plant species in Germany. Ecol. Evolution.

[54] Baubec T, Pecinka A, Rozhon W, Mittelsten Scheid O (2009). Effective, homogeneous and transient interference with cytosine methylation in plant genomic DNA by zebularine. Plant J..

[55] Ramos HCC, Pereira MG, Silva FF, Viana AP, Ferreguetti GA (2011). Seasonal and genetic influences on sex expression in a backcrossed segregating papaya population. Crop Breed. Appl. Biotechnol..

[56] Jiménez, V. M., Mora-Newcomer, E. & Gutiérrez-Soto, M. V. in *Genetics and Genomics of Papaya* (eds Ming, R. & Moore, P. H.) 17−33 (Springer, New York, 2014).

[57] Rodríguez Lorenzo JL, Hobza R, Vyskot B (2018). DNA methylation and genetic degeneration of the Y chromosome in the dioecious plant Silene latifolia. BMC Genomics.

[58] Bačovský Václav, Houben Andreas, Kumke Katrin, Hobza Roman (2019). The distribution of epigenetic histone marks differs between the X and Y chromosomes in Silene latifolia. Planta.

