# Supplementary material for: DNA methylome and transcriptome landscapes revealed differential characteristics of dioecious flowers in papaya
Source: Hortic Res. 2020 Jun 1;7:81. doi: 10.1038/s41438-020-0298-0 (PMC7261803; doi:10.1038/s41438-020-0298-0)
Supplement: Supplementary file 1 — Revised_manuscript_Supplementary_Figure 1.pdf [file 41438_2020_298_MOESM1_ESM.pdf]

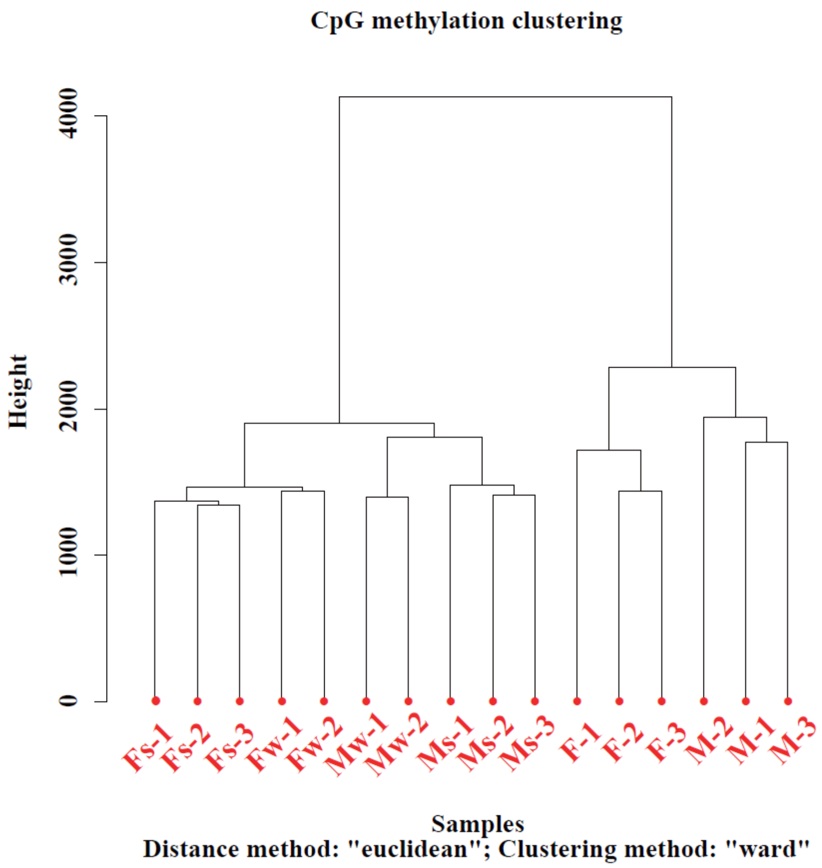
CpG methylation clustering
Height
4000
3000
2000
1000
0
Fs-1
Fs-2
Fs-3
Fw-1
Fw-2
Mw-1
Mw-2
Ms-1
Ms-2
Ms-3
F-1
F-2
F-3
M-2
M-1
M-3
Samples
Distance method: "euclidean"; Clustering method: "ward"

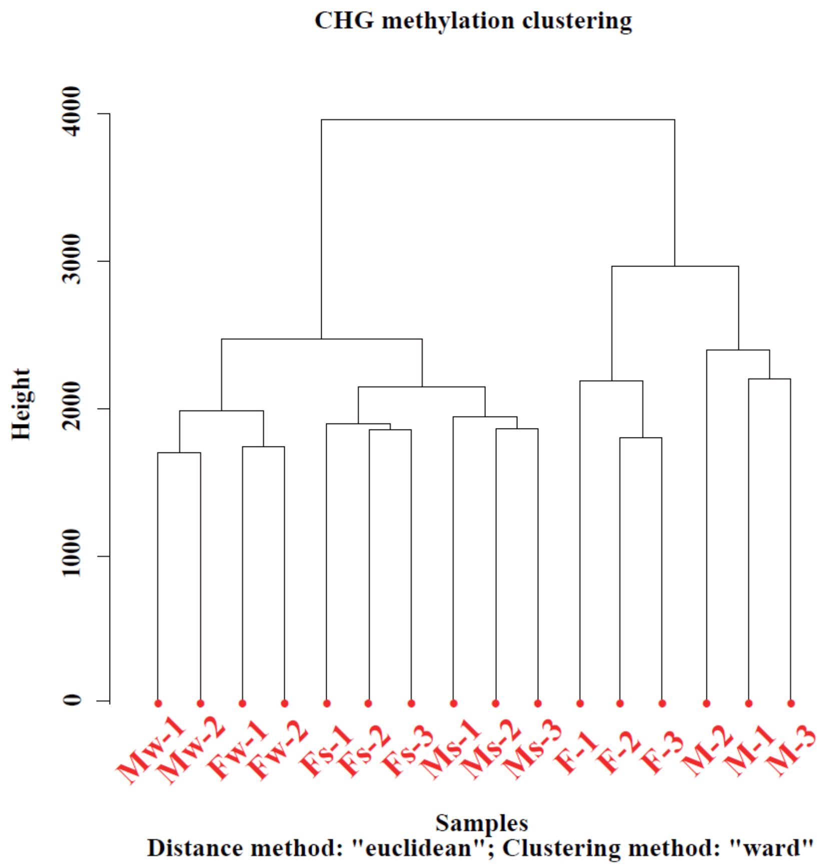
CHG methylation clustering
Height
4000
3000
2000
1000
0
Mw-1
Mw-2
Fw-1
Fw-2
Fs-1
Fs-2
Fs-3
Ms-1
Ms-2
Ms-3
F-1
F-2
F-3
M-2
M-1
M-3
Samples
Distance method: "euclidean"; Clustering method: "ward"

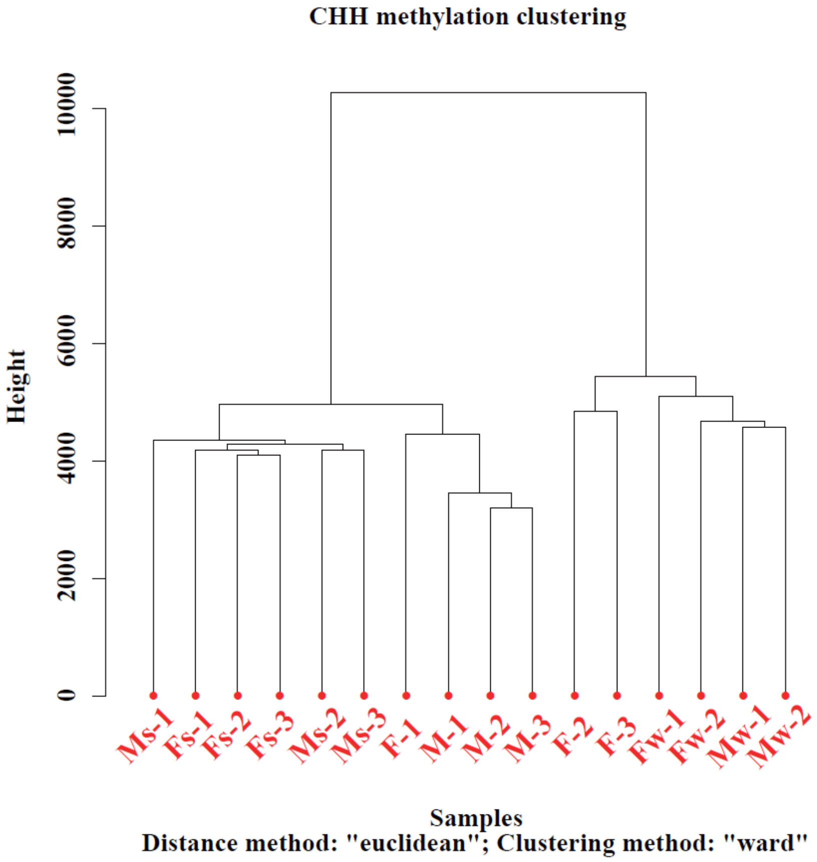

Supplementary Figure 1 Hierarchical clustering of 16 samples in CpG, CHG and CHH contexts
