# Supplementary material for: DNA methylome and transcriptome landscapes revealed differential characteristics of dioecious flowers in papaya
Source: Hortic Res. 2020 Jun 1;7:81. doi: 10.1038/s41438-020-0298-0 (PMC7261803; doi:10.1038/s41438-020-0298-0)
Supplement: Supplementary file 2 — Revised_manuscript_Supplementary_Figure 2.pdf [file 41438_2020_298_MOESM2_ESM.pdf]

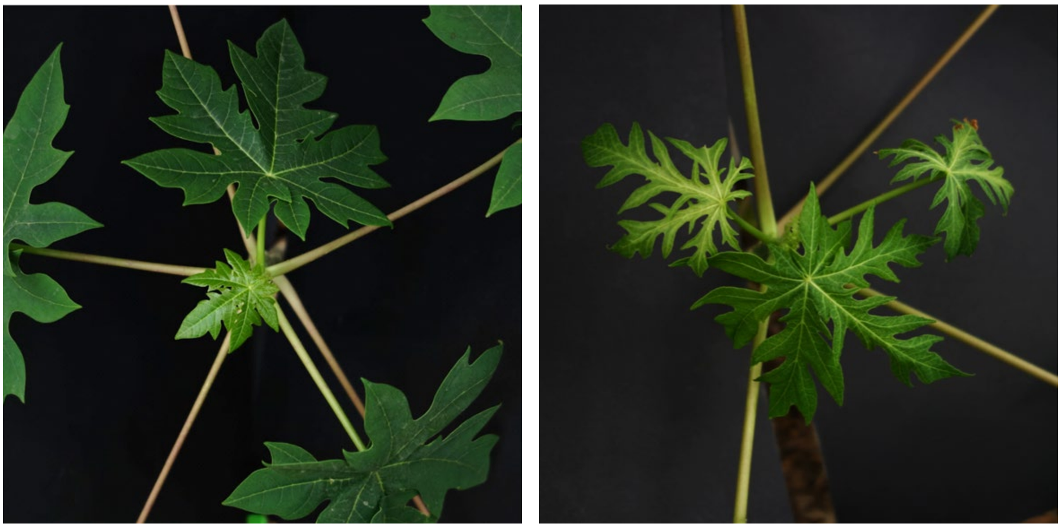

Supplementary Figure 2 Phenomenon of chemically hypo-methylation in papaya by DNA methylation inhibitor treatment. Graph (left) showed the papaya plant without Zebularine application; graph (right) was the plant with Zebularine treatment.
