# Supplementary material for: DNA methylome and transcriptome landscapes revealed differential characteristics of dioecious flowers in papaya
Source: Hortic Res. 2020 Jun 1;7:81. doi: 10.1038/s41438-020-0298-0 (PMC7261803; doi:10.1038/s41438-020-0298-0)
Supplement: Supplementary file 3 — Revised_manuscript_Supplementary_Table 1.pdf [file 41438_2020_298_MOESM3_ESM.pdf]

Supplementary Table 1 Summary of bisulfite sequencing data

Sample source	Sample names	Total number of sequence pairs	Theoretical sequencing depth	Total number of observed cytosines (at least 6 reads coverage)
Female flowers in spring	F1	181774064	155 ×	69459442
	F2	317916206	272 ×	71219007
	F3	311296521	226 ×	69020079
Male flowers in spring	M1	151320125	129 ×	89825685
	M2	136915437	117 ×	89680382
	M3	147989616	140 ×	89571835
Female flowers in summer	Fs1	112045989	96 ×	82178412
	Fs2	115728174	99 ×	83633781
	Fs3	128433938	110 ×	81300599
Male flowers in summer	Ms1	117559902	100 ×	82668346
	Ms2	138246727	118 ×	83308163
	Ms3	135706771	116 ×	83277450
Female flowers in winter	Fw1	132537436	113 ×	78714906
	Fw2	170492502	146 ×	82359777
Male flowers in winter	Mw1	146953101	125 ×	83508039
	Mw2	136659927	117 ×	82410448
