# Supplementary material for: DNA methylome and transcriptome landscapes revealed differential characteristics of dioecious flowers in papaya
Source: Hortic Res. 2020 Jun 1;7:81. doi: 10.1038/s41438-020-0298-0 (PMC7261803; doi:10.1038/s41438-020-0298-0)
Supplement: Supplementary file 4 — Revised_manuscript_Supplementary_Table 2.pdf [file 41438_2020_298_MOESM4_ESM.pdf]

Supplementary Table 2 The average methylation ratios of flower samples

Sample \ Sequence contexts	CpG	CHG	CHH
F-1（female flowers in spring）	79.1549 %	61.4239 %	5.9817 %
F-2（female flowers in spring）	80.3338 %	63.4217 %	8.9348 %
F-3（female flowers in spring）	79.9694 %	63.1497 %	9.2060 %
M-1（male flowers in spring）	79.7965 %	61.3563 %	5.6666 %
M-2（male flowers in spring）	78.8467 %	59.6708 %	3.9060 %
M-3（male flowers in spring）	79.4604 %	60.7333 %	4.9475 %
Fs-1（female flowers in summer）	81.6775 %	63.4453 %	6.0635 %
Fs-2（female flowers in summer）	81.6313 %	63.4238 %	6.0972 %
Fs-3（female flowers in summer）	81.7146 %	63.6039 %	6.2144 %
Ms-1（male flowers in summer）	81.4944 %	63.429 %	6.4303 %
Ms-2（male flowers in summer）	81.4941 %	63.5407 %	6.7509 %
Ms-3（male flowers in summer）	81.4510 %	63.4491 %	6.5899 %
Fw-1（female flowers in winter）	81.7120 %	64.9993 %	10.1820 %
Fw-2（female flowers in winter）	81.2076 %	64.3152 %	9.4026 %
Mw-1（male flowers in winter）	81.7541 %	64.7106 %	9.1560 %
Mw-2（male flowers in winter）	81.7844 %	64.7188 %	8.6955 %
