# Supplementary material for: DNA methylome and transcriptome landscapes revealed differential characteristics of dioecious flowers in papaya
Source: Hortic Res. 2020 Jun 1;7:81. doi: 10.1038/s41438-020-0298-0 (PMC7261803; doi:10.1038/s41438-020-0298-0)
Supplement: Supplementary file 5 — Revised_manuscript_Supplementary_Table 3.pdf [file 41438_2020_298_MOESM5_ESM.pdf]

Supplementary Table 3 The Pearson and Spearman correlation analyses of the methylated ratio differences of CsDMCs (in promoter regions) and the expressional value changes of downstream coding genes between male and female flowers across three seasons

	**M (F vs. M)**	**M (Fs vs. Ms)**	**M (Fw vs. Mw)**	**E (F vs. M)**	**E (Fs vs. Ms)**	**E (Fw vs. Mw)**
**M (F vs. M)**	-	0.76**	0.78**	0.06*	0.01	0.08**
**M (Fs vs. Ms)**	0.91**	-	0.87**	0.06*	-0.02	0.01
**M (Fw vs. Mw)**	0.92**	0.96**	-	0.07*	-0.04	0.02
**E (F vs. M)**	0.05	0.05	0.05	-	0.16**	0.05
**E (Fs vs. Ms)**	-0.01	-0.04	-0.04	0.02	-	0.04
**E (Fw vs. Mw)**	0.08**	0.02	0.03	0.05	-0.02	-

M (F vs. M), M (F vs. M) and M (F vs. M) represent methylated ratio differences of CsDMCs (in promoter regions) when comparing male and female flowers in spring, summer and winter, respectively. E (F vs. M), E (F vs. M) and E (F vs. M) represent the expressional value changes of downstream coding genes when comparing male and female flowers in spring, summer and winter, respectively. Correlation coefficient (Spearman's Rho) between two groups listed in the upper right of the table (see yellow cells), whereas correlation coefficient (Pearson's) in lower left of table (see grid cells). ** indicated $P < 0.01$, * indicated $P < 0.05$.
