# Supplementary material for: DNA methylome and transcriptome landscapes revealed differential characteristics of dioecious flowers in papaya
Source: Hortic Res. 2020 Jun 1;7:81. doi: 10.1038/s41438-020-0298-0 (PMC7261803; doi:10.1038/s41438-020-0298-0)
Supplement: Supplementary file 6 — Revised_manuscript_Supplementary_Table 4.pdf [file 41438_2020_298_MOESM6_ESM.pdf]

Supplementary Table 4 Details of methylation related gene expression patterns changes in different comparisons

Gene groups	Gene sub-groups		Gene names	Gene IDs in papaya genome	Fold Change of FPKM (log2 treated)						
					F(ref.) vs. M	Fs(ref.) vs. Ms	Fw(ref.) vs. Mw	F(ref.) vs. Fs	M(ref.) vs. Ms	F(ref.) vs. Fw	M(ref.) vs. Mw
Genes involved in DNA methylation	RNA-directed DNA methylation	(A1)	*CLSY2*	evm.TU.supercontig_19.123	0.222903	0.057999	-0.144529	0.312257	0.147353	-2.57368	-2.94111
		(A1)	*CLSY3*	evm.TU.supercontig_76.2	0.162676	0.422872	-0.547956	-0.485662	-0.225465	2.44571	1.73508
		(A1)	*SHH1a*	evm.TU.supercontig_109.7	0.0511542	-0.0613928	0.329879	-0.461107	-0.573654	-0.811019	-0.532294
		(A1)	*SHH1b*	evm.TU.supercontig_109.8	0.0511542	-0.0613928	0.329879	-0.461107	-0.573654	-0.811019	-0.532294
		(A1)	*RDR1a*	evm.TU.supercontig_2.176	0.240332	0.0755317	0.182465	0.910036	0.745236	0.0368228	-0.0210443
		(A1)	*RDR1b*	evm.TU.supercontig_2.177	0.240332	0.0755317	0.182465	0.910036	0.745236	0.0368228	-0.0210443
		(A1)	*RDR1c*	evm.TU.supercontig_2.178	0.240332	0.0755317	0.182465	0.910036	0.745236	0.0368228	-0.0210443
		(A1)	*RDR1d*	evm.TU.supercontig_2.183	0.868921	-0.0550928	-0.388036	1.34379	0.419775	2.26945	1.0125
		(A1)	*RDR2*	evm.TU.supercontig_124.30	-0.127821	0.00353407	0.347354	0.216032	0.347387	-0.639947	-0.164773
		(A1)	*RDR6*	evm.TU.supercontig_117.8	-0.125738	-0.151846	0.270544	0.161978	0.13587	-1.17944	-0.783157
		(A1)	*DCL1a*	evm.TU.supercontig_57.73	0.0101511	0.523963	-1.1932	-0.305402	0.20841	-1.79797	-3.00133
		(A1)	*DCL1b*	evm.TU.supercontig_57.74	-0.215135	-0.0665651	-1.21472	-0.42876	-0.28019	0.415174	-0.584411
		(A1)	*DCL2a*	evm.TU.supercontig_123.44	0.00260521	0.0699465	-0.463426	0.0852424	0.152584	0.197756	-0.268275
		(A1)	*DCL2b*	evm.TU.supercontig_123.45	0.00260521	0.0699465	-0.463426	0.0852424	0.152584	0.197756	-0.268275
		(A1)	*DCL2c*	evm.TU.supercontig_123.46	0.662487	0.305734	-2.28542	0.985323	0.62857	0.469004	-2.47891
		(A1)	*DCL3a*	evm.TU.supercontig_67.83	-0.0911995	0.000205171	-0.662909	0.215699	0.307104	0.33157	-0.240139
		(A1)	*DCL3b*	evm.TU.supercontig_67.84	-0.0911995	0.000205171	-0.662909	0.215699	0.307104	0.33157	-0.240139
		(A1)	*DCL3c*	evm.TU.supercontig_67.86	-0.0911995	0.000205171	-0.662909	0.215699	0.307104	0.33157	-0.240139
		(A1)	*DCL3d*	evm.TU.contig_40955	-0.200059	0.115332	-1.02594	0.189449	0.50484	0.745542	-0.0803384
		(A1)	*DCL4a*	evm.TU.supercontig_397.1	-0.220791	-0.132428	-1.17856	-1.2395	-1.15114	-0.521723	-1.47949
		(A1)	*DCL4b*	evm.TU.supercontig_397.3	-0.220791	-0.132428	-1.17856	-1.2395	-1.15114	-0.521723	-1.47949
		(A1)	*DCL4c*	evm.TU.supercontig_397.5	-0.220791	-0.132428	-1.17856	-1.2395	-1.15114	-0.521723	-1.47949
		(A1)	*DCL4d*	evm.TU.supercontig_46.92	-0.0674471	-0.973998	-2.28397	0.000208075	-0.906343	-0.616515	-2.83304
		(A1)	*DCL4e*	evm.TU.supercontig_46.94	-0.0674471	-0.973998	-2.28397	0.000208075	-0.906343	-0.616515	-2.83304
		(A1)	*DCL4f*	evm.TU.contig_36105	-0.238015	0.307108	-1.5688	-0.382004	0.16312	-0.567196	-1.89798
		(A1)	*AGO1a*	evm.TU.supercontig_47.31	-0.017164	0.185112	-0.304895	-0.178971	0.0233059	-0.522469	-0.8102
		(A1)	*AGO1b*	evm.TU.supercontig_1673.2	-0.105708	0.332502	-0.0162811	-3.45722	-3.01901	-4.1991	-4.10967
		(A1)	*AGO1c*	evm.TU.contig_46512	-0.0825227	0.0593581	-0.100069	-0.28112	-0.139239	-0.877655	-0.895201
		(A1)	*AGO2a*	evm.TU.contig_41691	0.561495	-0.64171	-0.288094	2.99853	1.79532	4.79075	3.94117
		(A1)	*AGO2b*	evm.TU.supercontig_135.40	0.138163	0.266554	-0.168588	-0.241519	-0.113127	-0.247947	-0.554697
		(A1)	*AGO4*	evm.TU.supercontig_26.59	-0.146807	0.194488	-0.133234	0.168624	0.509919	-1.02817	-1.0146
		(A1)	*AGO7*	evm.TU.supercontig_1.68	0.290224	0.673197	0.602845	-0.526087	-0.143114	-1.61368	-1.30105
		(A1)	*AGO9*	evm.TU.supercontig_600.1	-0.120319	0.157126	-0.0761734	-0.441019	-0.163574	-1.37713	-1.33298
		(A1)	*AGO10a*	evm.TU.supercontig_44.130	0.0658198	0.0663537	0.109275	0.283885	0.284419	-1.33383	-1.29037
		(A1)	*AGO10b*	evm.TU.supercontig_75.90	-0.0795312	0.209986	-0.0440624	-0.477173	-0.187656	-2.21216	-2.17669
		(A1)	*DRM1*	evm.TU.supercontig_373.3	-0.459297	-0.291194	0.0749376	1.261	1.4291	-1.27112	-0.736884
		(A1)	*DRM2*	evm.TU.supercontig_470.2	0.11496	0.167496	-0.0714336	0.196904	0.24944	0.707488	0.521094
		(A1)	*DRM3a*	evm.TU.supercontig_146.49	-0.253028	0.191413	-0.650644	-0.175728	0.268713	-1.77891	-2.17652
		(A1)	*DRM3b*	evm.TU.supercontig_146.50	0.178506	0.0364564	0.0949943	1.10119	0.959144	0.409859	0.326348
		(A1)	*IDN1*	evm.TU.supercontig_39.5	-0.12703	-0.508418	0.0935065	0.156139	-0.225249	-1.80125	-1.58072
		(A1)	*IDN2*	evm.TU.supercontig_1.418	-0.138454	-0.191104	0.517152	0.745404	0.692755	-0.702138	-0.0465318
		(A1)	*IDP1*	evm.TU.supercontig_10.88	0.0851183	-0.0135877	0.479807	0.254687	0.155981	-1.07871	-0.684022
		(A1)	*RRP6L1*	evm.TU.supercontig_87.78	-0.305657	0.0294954	-0.0337534	0.265048	0.6002	-0.524799	-0.252895
	Maintenance of DNA methylation	(A2)	*MET1*	evm.TU.supercontig_37.192	0.0147706	0.0802334	0.181334	-0.940436	-0.874973	-2.18024	-2.01367
		(A2)	*CMT2a*	evm.TU.contig_46763	-0.480434	1.04118	0.539795	0.22783	1.74945	-3.33891	-2.31868

		(A2)	*CMT2b*	evm.TU.supercontig_133.1	0.00291933	0.0929509	0.225786	0.299356	0.389388	-2.8523	-2.62944
		(A2)	*CMT3c*	evm.TU.supercontig_27.168	-0.26165	-0.269973	3.22585	-0.905841	-0.914163	-7.41744	-3.92994
		(A2)	*CMT3d*	evm.TU.supercontig_48.151	0.0163085	-0.305546	-0.0169025	0.730725	0.40887	-0.0794022	-0.112613
		(A2)	*DRM2*	evm.TU.supercontig_470.2	0.11496	0.167496	-0.0714336	0.196904	0.24944	0.707488	0.521094
		(A2)	*SUVH4*	evm.TU.supercontig_70.67	-0.333211	-0.116595	0.567708	0.311237	0.527853	-1.98852	-1.0876
		(A2)	*SUVH6*	evm.TU.contig_35939	-0.0822372	0.125532	-0.509828	-0.581063	-0.373293	-0.0405522	-0.468143
	DNA Demethylation	(A3)	*ROS1*	evm.TU.supercontig_5.80	0.14876	0.186602	-0.121938	0.223845	0.261687	-1.26863	-1.53933
		(A3)	*DME*	evm.TU.supercontig_128.52	-0.146997	-0.34125	-1.70739	-1.28743	-1.48169	-1.27444	-2.83483
		(A3)	*DME*	evm.TU.supercontig_5.81	-0.262736	0.300617	-0.37133	-1.01632	-0.452968	-1.78111	-1.88971
		(A3)	*ZDP*	evm.TU.supercontig_77.9	-0.122353	-0.0604196	0.562896	-0.0688937	-0.00696067	-1.00298	-0.317734
		(A3)	*APE*	evm.TU.supercontig_6.256	-0.119182	0.0212076	-0.212314	-0.488455	-0.348066	0.495579	0.402446
		(A3)	*LIG1a*	evm.TU.supercontig_1.10	0.318382	0.473684	-0.630409	-0.601462	-0.44616	0.156836	-0.791955
		(A3)	*LIG1b*	evm.TU.supercontig_1.8	-0.152254	0.358898	-0.495869	-1.03464	-0.523488	-1.46475	-1.80836
		(A3)	*IDM1*	evm.TU.supercontig_97.95	-0.158739	-0.0665913	-0.698226	-0.509878	-0.41773	-0.498697	-1.03818
		(A3)	*HDP1*	evm.TU.supercontig_29.100	-0.224091	-0.058818	-0.220191	0.507515	0.672789	0.59378	0.59768
		(A3)	*HDP2*	evm.TU.supercontig_148.5	0.112471	-0.0153484	0.249771	0.522036	0.394216	0.451373	0.588672
		(A3)	*IDM2*	evm.TU.supercontig_48.29	0.0312353	0.273953	-0.145973	-0.947343	-0.704625	0.824086	0.646878

Genes involved in histone modification	Histone Chaperone HIRA	(B1)	*HIRAa*	evm.TU.contig_36505.3	-0.0692791	0.196046	-0.159362	-0.318873	-0.053548	1.0282	0.938122
		(B1)	*HIRAb*	evm.TU.supercontig_1624.1	-0.160893	0.0363554	-0.499724	-0.72444	-0.527192	-0.226323	-0.565154
	Histone Monobiquitination 1- like	(B2)	*HUB1a*	evm.TU.supercontig_101.40	0.117996	0.0591987	-0.831151	-0.0174307	-0.0762282	0.801882	-0.147266
		(B2)	*HUB1b*	evm.TU.supercontig_101.41	0.117996	0.0591987	-0.831151	-0.0174307	-0.0762282	0.801882	-0.147266
		(B2)	*HUB2a*	evm.TU.supercontig_119.82	-0.330648	-0.437073	-0.0976753	1.431	1.32458	0.783184	1.01616
		(B2)	*HUB2b*	evm.TU.supercontig_119.83	-0.330648	-0.437073	-0.0976753	1.431	1.32458	0.783184	1.01616
	Histone deacetylase	(B3)	*HDA1a*	evm.TU.contig_48086	0.0901894	0.243667	-0.496475	-0.891723	-0.738245	-0.12787	-0.714535
		(B3)	*HDA1b*	evm.TU.supercontig_63.33	-0.50181	0.184288	-1.20415	-2.76374	-2.07764	-0.713774	-1.41612
		(B3)	*HDA1c*	evm.TU.supercontig_63.37	-0.50181	0.184288	-1.20415	-2.76374	-2.07764	-0.713774	-1.41612
		(B3)	*HDA2*	evm.TU.supercontig_7.166	-0.0753456	-0.0627526	0.625826	0.644375	0.656968	1.13321	1.83438
		(B3)	*HD2Ca*	evm.TU.supercontig_47.60	-0.272008	-0.128737	-0.172456	0.219675	0.362946	0.787273	0.886825
		(B3)	*HD2Cb*	evm.TU.supercontig_5.85	-0.0769227	-0.204628	0.119981	0.385867	0.258162	0.542151	0.739055
		(B3)	*HD2Cc*	evm.TU.supercontig_599.1	0.0301759	-0.0840347	0.0664008	-0.245299	-0.35951	-0.464154	-0.427929
		(B3)	*HDA5a*	evm.TU.supercontig_140.1	0.108016	0.106548	-0.502243	0.390673	0.389205	1.73896	1.12871
		(B3)	*HDA5b*	evm.TU.supercontig_140.11	0.342949	0.275768	-0.267244	-0.597706	-0.664887	0.984726	0.374534
		(B3)	*HDA5c*	evm.TU.supercontig_140.13	-0.00627715	0.0460774	0.255876	-0.384465	-0.33211	1.89642	2.15857
		(B3)	*HDA5d*	evm.TU.supercontig_67.15	1.55995	-1.31137	-1.0702	2.90902	0.0377005	2.31176	-0.318395
		(B3)	*HDA5e*	evm.TU.supercontig_94.49	0.155486	-0.0284847	0.387543	-0.325771	-0.509742	-0.249833	-0.0177759
		(B3)	*HDA5f*	evm.TU.supercontig_94.50	0.155486	-0.0284847	0.387543	-0.325771	-0.509742	-0.249833	-0.0177759
		(B3)	*HDA5g*	evm.TU.supercontig_99.2	0.383766	0.97257	-0.0127759	0.0588738	0.647678	1.88706	1.49052
		(B3)	*HDA6*	evm.TU.supercontig_110.13	-0.321186	-0.131275	0.246349	0.627622	0.817533	0.0886111	0.656146
		(B3)	*HDA8*	evm.TU.supercontig_754.5	-0.132133	-0.31585	-0.694976	1.81123	1.62751	0.61142	0.0485771
		(B3)	*HDA9a*	evm.TU.supercontig_31.89	0.160058	-0.0492313	-0.47042	0.47748	0.26819	0.889219	0.25874
		(B3)	*HDA9b*	evm.TU.supercontig_509.2	-0.168405	-0.184423	-0.663028	-0.587666	-0.603685	-0.373168	-0.867792
		(B3)	*HDA14*	evm.TU.supercontig_42.168	-0.130452	0.0168971	-1.05996	0.516852	0.664201	0.297937	-0.631568
		(B3)	*HDA15*	evm.TU.supercontig_13.45	-0.333876	-0.288364	0.169873	0.427881	0.473394	-0.506118	-0.00236957
		(B3)	*HDA18a*	evm.TU.contig_36738	0.284623	-0.0561281	-1.01543	-0.313615	-0.654366	1.53502	0.234972
		(B3)	*HDA18b*	evm.TU.supercontig_67.16	-0.497077	0.16392	-0.352709	-0.230863	0.430134	0.78923	0.933598
		(B3)	*HDA18c*	evm.TU.supercontig_99.1	0.383766	0.97257	-0.0127759	0.0588738	0.647678	1.88706	1.49052
		(B3)	*HDA18d*	evm.TU.supercontig_99.3	0.383766	0.97257	-0.0127759	0.0588738	0.647678	1.88706	1.49052

	Histone acetyltransferase	(B4)	*HAC1a*	evm.TU.supercontig_10.17	-0.173295	0.149393	-1.37163	-0.227923	0.0947653	0.240304	-0.958033
		(B4)	*HAC1b*	evm.TU.supercontig_818.3	-0.376582	-0.255035	-1.17798	1.02619	1.14774	2.50133	1.69993
		(B4)	*HAC1c*	evm.TU.supercontig_179.37	-0.377152	-0.383332	-1.23016	0.748553	0.742372	1.75525	0.902244
		(B4)	*HAG1*	evm.TU.supercontig_52.102	-0.433676	-0.0428055	-0.530901	0.415136	0.806006	-0.154445	-0.25167
		(B4)	*HAG2*	evm.TU.supercontig_32.101	-0.0679572	0.0226659	-0.0300333	-0.953174	-0.86255	-0.0236615	0.0142623
		(B4)	*HAM1*	evm.TU.supercontig_42.141	0.0820798	-0.00748923	0.0262313	0.301677	0.212108	0.179067	0.123218
		(B4)	*HAM2*	evm.TU.supercontig_42.140	0.0820798	-0.00748923	0.0262313	0.301677	0.212108	0.179067	0.123218
	SET-domain group protein	(B5)	*SDG7*	evm.TU.supercontig_9.234	-0.0454953	-0.275642	-0.656991	0.329467	0.0993201	0.473281	-0.138215
		(B5)	*SDG8*	evm.TU.supercontig_26.315	-0.0133014	0.0736446	-1.02415	-0.499482	-0.412536	-0.0236889	-1.03454
		(B5)	*SDG26*	evm.TU.supercontig_49.86	0.0236377	-0.0787096	-0.295975	0.0829444	-0.019403	-0.00149312	-0.321106
		(B5)	*SDG36*	evm.TU.supercontig_8.235	0.18054	0.287485	-1.31133	0.246541	0.353486	1.14921	-0.342661

Genes involved in chromatin remodeling	Chromatin remodeling		*IDN1*	evm.TU.supercontig_39.5	-0.12703	-0.508418	0.0935065	0.156139	-0.225249	-1.80125	-1.58072
			IDP1	evm.TU.supercontig_10.88	0.0851183	-0.0135877	0.479807	0.254687	0.155981	-1.07871	-0.684022
			FDM4a	evm.TU.supercontig_81.135	-0.325537	-0.409065	-0.0563799	1.21991	1.13638	-0.0918898	0.177267
			FDM4b	evm.TU.supercontig_81.136	-0.325537	-0.409065	-0.0563799	1.21991	1.13638	-0.0918898	0.177267
			SWI3Aa	evm.TU.contig_40216	0.160966	0.102649	0.165329	0.151652	0.0933356	0.415035	0.419398
			SWI3Ab	evm.TU.supercontig_4.159	0.00212063	0.236042	-0.557991	0.0320351	0.265957	1.27521	0.715103
			SWI3Ac	evm.TU.supercontig_4.161	0.00212063	0.236042	-0.557991	0.0320351	0.265957	1.27521	0.715103
			SWI3B	evm.TU.supercontig_26.13	0.042149	0.0494761	-0.104629	-0.222038	-0.214711	0.495052	0.348273
			SWI3Ca	evm.TU.supercontig_64.124	0.0305236	-0.0769048	0.190936	0.624531	0.517102	0.847536	1.00795
			SWI3Cb	evm.TU.supercontig_64.126	0.0305236	-0.0769048	0.190936	0.624531	0.517102	0.847536	1.00795
			SWI3Cc	evm.TU.supercontig_64.127	0.0305236	-0.0769048	0.190936	0.624531	0.517102	0.847536	1.00795
			SWI3D	evm.TU.supercontig_62.137	-0.145152	0.36808	-0.495699	-0.421198	0.0920347	-0.422006	-0.772553
			SNF2-like1	evm.TU.contig_38871	-0.0773177	-0.00904353	-1.30891	0.1144	0.182674	0.66038	-0.57121
			SNF2-like2	evm.TU.supercontig_221.20	-0.0612522	-0.0981509	-0.55319	0.352368	0.315469	1.619	1.12706
			SNF2-like3	evm.TU.contig_36046	0.1074	0.3474	0.2168	0.1156	0.2136	0.2136	0.3146
			SNF2-like4	evm.TU.contig_38871	-0.0773177	-0.00904353	-1.30891	0.1144	0.182674	0.66038	-0.57121
			SNF2-like5	evm.TU.contig_44154	0.00651491	0.0129277	-0.818084	0.39935	0.405763	1.41668	0.592081
			SNF2-like6	evm.TU.supercontig_221.20	-0.0612522	-0.0981509	-0.55319	0.352368	0.315469	1.619	1.12706
			SNF2-like7	evm.TU.supercontig_2800.1	0.617066	-0.366804	-0.307794	1.79626	0.812388	1.94994	1.02508
			SNF2-like8	evm.TU.supercontig_94.1	-0.0429711	-0.0706649	-0.372264	0.355906	0.328212	-0.0654889	-0.394781
			SNF2-like9	evm.TU.supercontig_997.1	-0.0333021	-0.0666798	-1.52599	-0.022311	-0.0556887	0.431486	-1.06121
			INO80	evm.TU.supercontig_182.16	-0.0264075	-0.0488095	-0.166547	0.265503	0.243101	-0.00201199	-0.142151
			ARP6	evm.TU.supercontig_46.24	-0.165939	-0.120253	-0.13283	0.702787	0.748473	0.263365	0.296474
			CHC1a	evm.TU.supercontig_13.130	0.114426	0.0653091	-0.0437897	-0.230237	-0.279354	0.52085	0.362635
			CHC1b	evm.TU.supercontig_171.24	-0.0444239	0.0138418	-0.483925	0.44677	0.505036	1.02532	0.585822
			HR(B2)	evm.TU.supercontig_127.15	-0.143556	0.00791456	-0.191422	-0.712	-0.56053	-0.447682	-0.495548
			CHR2/BRAHMAa	evm.TU.supercontig_11.23	-0.37175	-0.514798	-0.550304	0.0138174	-0.12923	-2.1412	-2.31976
			CHR2/BRAHMAb	evm.TU.supercontig_11.24	-0.547841	-0.0946829	-0.563702	0.504899	0.958058	-0.135614	-0.151475
			CHR3/SYDa	evm.TU.supercontig_16.99	-0.115753	0.0197376	-0.705347	-0.168412	-0.0329214	0.0297712	-0.559823
			CHR3/SYDb	evm.TU.supercontig_16.94	-0.233292	-0.0965124	0.00317027	-0.526783	-0.390004	0.0632281	0.29969
			CHR3/SYDc	evm.TU.supercontig_16.100	-0.233292	-0.0965124	0.00317027	-0.526783	-0.390004	0.0632281	0.29969
			CHR3/SYDd	evm.TU.supercontig_16.103	-0.233292	-0.0965124	0.00317027	-0.526783	-0.390004	0.0632281	0.29969
			CHR4/PKR1	evm.TU.supercontig_30.142	-0.181953	-0.029815	-0.855681	-0.429284	-0.277146	-0.0295481	-0.703276
			CHR5a	evm.TU.contig_34800	0	0	0	0	0	0	0
			CHR5b	evm.TU.contig_36207	0.0692654	-0.018602	-1.56511	-0.431208	-0.519076	0.308321	-1.32605
			CHR5c	evm.TU.contig_37405	-0.0705122	0.09131	-1.99177	-0.650464	-0.488642	-0.460642	-2.3819

		CHR5d	evm.TU.supercontig_84.18	-0.0598339	0.138014	-1.92293	-1.12082	-0.922969	-1.40523	-3.26833
		CHR5e	evm.TU.supercontig_892.1	-0.038926	-0.197975	-0.714114	-0.237326	-0.396375	0.990946	0.315758
		CHR6/PICKLE	evm.TU.supercontig_127.15	-0.143556	0.00791456	-0.191422	-0.712	-0.56053	-0.447682	-0.495548
		CHR8	evm.TU.supercontig_165.16	0.210787	-0.124492	-0.586915	0.662343	0.327064	1.98338	1.18568
		CHR9	evm.TU.supercontig_83.60	0.0190018	0.229193	-0.862049	0.503015	0.713206	0.878914	-0.00213749
		CHR11	evm.TU.supercontig_16.129	0.118406	0.114274	0.130151	-0.0269921	-0.0311238	0.489266	0.501011
		CHR12a	evm.TU.supercontig_1278.1	-0.116321	-0.249236	-0.0692929	0.693914	0.561	0.80413	0.851159
		CHR12b	evm.TU.contig_45714	-0.33925	-0.086244	-0.981142	0.142707	0.395713	-1.09676	-1.73865
		CHR13/PIE1/SRCAPa	evm.TU.contig_33695	-0.495237	0.147611	-1.48528	-0.464122	0.178726	-1.61096	-2.601
		CHR13/PIE1/SRCAPb	evm.TU.contig_39138	-0.460475	1.73176	-2.89535	-1.33401	0.858224	-0.867715	-3.30259
		CHR13/PIE1/SRCAPc	evm.TU.supercontig_34.19	-0.28412	-0.0331593	-0.797035	0.176979	0.42794	0.201507	-0.311408
		CHR13/PIE1/SRCAPd	evm.TU.supercontig_34.20	-0.28412	-0.0331593	-0.797035	0.176979	0.42794	0.201507	-0.311408
		CHR18a	evm.TU.supercontig_146.11	-0.131142	-0.159268	-0.509333	0.608791	0.580666	-0.0825961	-0.460787
		CHR18b	evm.TU.supercontig_146.12	-0.131142	-0.159268	-0.509333	0.608791	0.580666	-0.0825961	-0.460787
		CHR19	evm.TU.contig_26556.2	-0.0789214	-0.0389437	-0.699851	-0.570285	-0.530307	-0.0385973	-0.659527
		CHR24a	evm.TU.contig_40991	-0.110076	-0.103686	-0.649151	-1.29081	-1.28442	-4.03701	-4.57609
		CHR24b	evm.TU.supercontig_1639.3	-0.113816	-0.566615	-0.331423	-0.590584	-1.04338	-2.26481	-2.48242
		CHR31/CLSY3	evm.TU.supercontig_76.2	0.162676	0.422872	-0.547956	-0.485662	-0.225465	2.44571	1.73508
		CHR35/DMS1/DRD1	evm.TU.supercontig_1026.2	-0.237272	0.181321	-1.14466	-0.61858	-0.199987	0.93868	0.0312969
		CHR42/CLSY2a	evm.TU.supercontig_19.122	0.222903	0.057999	-0.144529	0.312257	0.147353	-2.57368	-2.94111
		CHR42/CLSY2b	evm.TU.supercontig_19.123	0.222903	0.057999	-0.144529	0.312257	0.147353	-2.57368	-2.94111
		CHR-like1	evm.TU.supercontig_132.8	-0.273388	-0.261747	0.0764495	0.502865	0.514505	-0.275693	0.0741443
		CHR-like2	evm.TU.supercontig_152.54	-0.191894	-0.503098	-0.0893474	1.09629	0.785085	-0.61397	-0.511424
		CHR-like3	evm.TU.supercontig_1026.2	-0.237272	0.181321	-1.14466	-0.61858	-0.199987	0.93868	0.0312969
		MOM1a	evm.TU.supercontig_311.2	-0.102047	-0.0281104	-0.587811	0.0451466	0.119084	0.193031	-0.292733
		MOM1b	evm.TU.supercontig_311.4	-0.102047	-0.0281104	-0.587811	0.0451466	0.119084	0.193031	-0.292733
		IBM1	evm.TU.supercontig_707.1	-0.222791	-0.0730116	-0.361288	-0.700303	-0.550524	-0.744736	-0.883234
		SYN4 in SDR	CpXY8	0.48082397	1.577373408	0.073752298	-1.23468329	0.091471235	-0.13813385	-0.315600437
		MBD9 in SDR	CpXY20	-0.822931	-1.14172	-2.38812	0.374874	-0.616345	0.0560815	-2.18153
