# Supplementary material for: DNA methylome and transcriptome landscapes revealed differential characteristics of dioecious flowers in papaya
Source: Hortic Res. 2020 Jun 1;7:81. doi: 10.1038/s41438-020-0298-0 (PMC7261803; doi:10.1038/s41438-020-0298-0)
Supplement: Supplementary file 7 — Revised_manuscript_Supplementary_Table 5.pdf [file 41438_2020_298_MOESM7_ESM.pdf]

Supplementary Table 5. The expression levels of 11 sex-associated DEGs involved in phytohormone signal transduction

		Gene names	Gene IDs	Homologous genes in *Arabidopsis*	Expression levels of samples (FKPM)					
					F	M	Fs	Ms	Fw	Mw
Auxin signaling:	AUX	*CpAUX1*	evm.TU.supercontig_1125.1	AT2G38120 (*AtAUX1*)	23.7948	46.1924	20.8324	37.2311	3.76457	32.3549
	TIR/AFB	*CpTIR1*	evm.TU.supercontig_27.130	AT3G62980 (*AtTIR1*)	60.3436	85.5193	54.3516	78.7706	12.915	31.9012
	ARF5	*CpARF5*	evm.TU.supercontig_26.24	AT1G19850 (*AtARF5*)	215.989	321.318	204.633	382.788	40.575	74.4848
	IAA4	*CpIAA4*	evm.TU.supercontig_58.36	AT5G43700 (*AtIAA4*)	42.4561	96.0638	45.5219	73.4007	19.7624	263.782
	IAA16	*CpIAA16*	evm.TU.supercontig_23.159	AT3G04730 (*AtIAA16*)	10.4961	21.8751	11.0651	31.9072	14.4097	199.08
Cytokinine signaling:	AHP1	*CpAHP1*	evm.TU.supercontig_84.120	AT3G21510 (*AtAHP1*)	90.7504	12.8406	162.069	10.4044	161.647	31.5317
	ARR4	*CpARR4*	evm.TU.supercontig_5.328	AT1G10470 (*AtARR4*)	31.9291	19.0782	48.1832	28.48	67.1488	39.7917
	ARR5	*CpARR5*	evm.TU.supercontig_35.27	AT3G48100 (*AtARR5*)	91.692	39.4026	72.5142	31.9628	215.258	62.5166
ABA signaling:	HAI2	*Cp HAI2*	evm.TU.supercontig_81.90	AT1G07430 (*AtHAI2*)	246.229	101.681	636.421	234.848	491.063	57.9741
	SNRK2	*CpSnRK2*	evm.TU.supercontig_36.167	AT1G78290 (*AtSNRK2.8*)	26.0302	15.0888	30.0506	19.0755	47.5324	21.5901
Ethyene signaling:	EIN3	*CpEIN3*	evm.TU.supercontig_54.30	AT3G20770 (*AtEIN3*)	24.4558	10.8401	55.9213	27.0891	70.3478	30.976
