# Supplementary material for: DNA methylome and transcriptome landscapes revealed differential characteristics of dioecious flowers in papaya
Source: Hortic Res. 2020 Jun 1;7:81. doi: 10.1038/s41438-020-0298-0 (PMC7261803; doi:10.1038/s41438-020-0298-0)
Supplement: Supplementary file 8 — Revised_manuscript_Supplementary_Table 6.pdf [file 41438_2020_298_MOESM8_ESM.pdf]

Supplementary Table 6. Ten sex-assocaited genes harboring CsDMCs

10 sex-assocaited genes harboring CsDMCs		CsDMCs location (contig, position)		Gene regions where containing CsDMCs	[illegible] samples comparing to female ones
CpARR5	(evm.TU.supercontig_35.27)	supercontig_35	582774	downstream	hypo
CpACP4	(evm.TU.supercontig_16.107)	supercontig_16	1650440	downstream	hypo
		supercontig_16	1650483	downstream	hypo
		supercontig_16	1650494	downstream	hypo
		supercontig_16	1650702	introns_3	hyper
		supercontig_16	1650965	introns_3	hypo
CpAPE2	(evm.TU.supercontig_27.1)	supercontig_27	3873	promoter	hypo
		supercontig_27	3879	promoter	hypo
		supercontig_27	3894	promoter	hypo
		supercontig_27	3896	promoter	hypo
		supercontig_27	3916	promoter	hypo
CpGsSRK	(evm.TU.supercontig_12.8)	supercontig_12	35657	downstream	hypo
CpMAGL4	(evm.TU.supercontig_3.339)	supercontig_3	2346636	downstream	hyper
		supercontig_3	2346663	downstream	hyper
		supercontig_3	2346896	downstream	hypo
		supercontig_3	2349395	introns_3	hypo
		supercontig_3	2349563	introns_3	hypo
		supercontig_3	2349585	introns_3	hypo
		supercontig_3	2351055	introns_3	hyper
Cp NB-ARC	(evm.TU.supercontig_27.143)	supercontig_27	1577031	promoter	hypo
		supercontig_27	1577296	promoter	hyper
		supercontig_27	1577333	promoter	hyper
CpSBT3.3	(evm.TU.supercontig_285.9)	supercontig_285	65031	exons_4	hypo
CpTFIID	(evm.TU.supercontig_14.189)	supercontig_14	2119080	exons_1	hypo
CpTLC	(evm.TU.supercontig_99.8)	supercontig_99	114934	downstream	hyper
		supercontig_99	118204	promoter	hypo
CpUNC	(evm.TU.supercontig_14.197)	supercontig_14	2180370	downstream	hypo
